# Assessing nature-based coastal defense

**DOI:** 10.1038/s41598-025-96744-7

**Published:** 2025-05-14

**Authors:** Virginie K. E. Duvat, Inès Hatton, Louise Burban, Alice Jacobée, Myriam Vendé-Leclerc, Lucile Stahl

**Affiliations:** 1 UMRi LIENSs 7266, La Rochelle Université-CNRS, 2 rue Olympe de Gouges, 17000 La Rochelle, France; 2 Cawthron Institute, 98 Halifax Street, Nelson, 7010 New Zealand; 3 Observatoire du Littoral de Nouvelle-Calédonie/DIMENC, 1 ter Rue Unger, Nouméa, New Caledonia; 4 Service de la Météorologie de Nouvelle-Calédonie, 5 rue Vincent Auriol, Faubourg Blanchot, Nouméa, New Caledonia

**Keywords:** Ecosystem-based adaptation, Nature-based solutions, Coastal risk reduction, Structured expert judgment, Climate change, Small Islands, Climate-change adaptation, Climate-change policy, Environmental impact, Sustainability

## Abstract

Nature-based Coastal Defense is increasingly used to reduce climate risk, because considered effective, inexpensive and cost-effective, easy to implement, and no-regrets. This article discusses this positive framing through the analysis of 23 projects implemented in French overseas territories, using an *ex-post* expert judgment method considering enabling conditions (context, governance, funding, social acceptability), risk reduction (technical effectiveness; studies, monitoring and evaluation) and externalities (co-benefits and disbenefits; contribution to adaptation). 80% of projects aimed at reducing coastal erosion; 47.8% were implemented in natural or rural areas; 87.1% included restoration; 82.6% targeted one single ecosystem and 51.7% beach/dune systems; 47.8% were led by public actors; all relied on multiple funding sources. Performance indices range from 39.4 to 77.2%. The highest scores concern governance and social acceptability, and the lowest scores risk reduction. No project included an evaluation of risk reduction and was calibrated for future risk. Internal (i.e. project related) and external (more general) levers and barriers to effectiveness were identified. Internal barriers include the lack of political support to nature-based options, the difficulty to secure the required long-term funding and to upscale action in the face of strong land tenure constraints.

**Supplementary Information:**

The online version contains supplementary material available at 10.1038/s41598-025-96744-7.

## Introduction

**Fig. 1:**
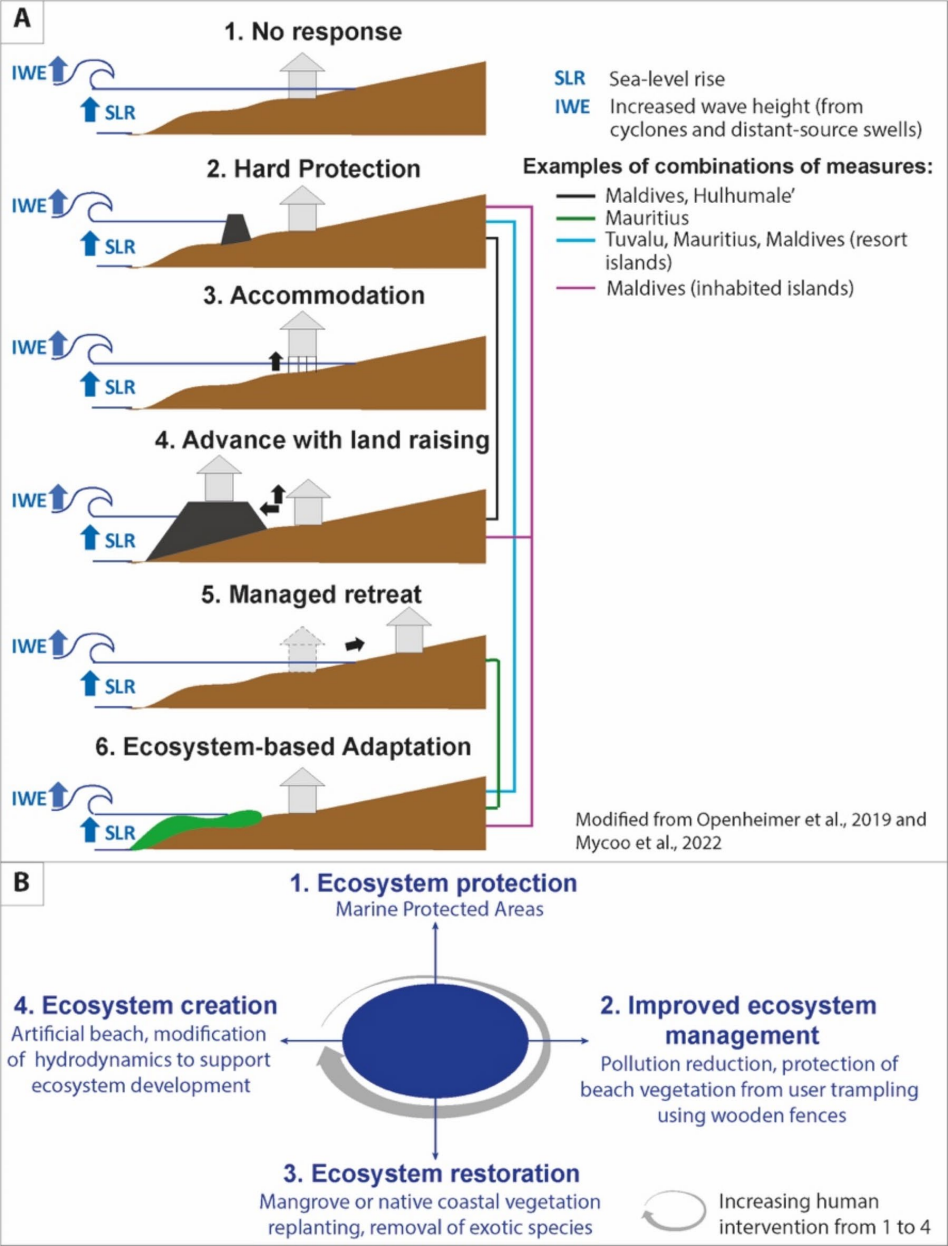
Categorization of coastal adaptation measures. (**A**) Shows the 6 main types of coastal adaptation measures and provides selected island examples of combinations of measures. (**B**) Highlights the 4 main types of NbCD measures, with key examples. It shows increasing human intervention from ecosystem protection (Type 1) to ecosystem creation (Type 4).

In the face of accelerating climate change, island territories implement a variety of coastal risk reduction and climate adaptation measures^1^. According to the IPCC^2^, the latter include ‘do nothing’ (no intervention in the face of risks), hard protection (by engineered structures), accommodation (e.g. promotion of flood-proof buildings), advance with land raising (settling artificial land higher than natural areas), managed retreat (landward relocation of threatened assets), and Ecosystem-based Adaptation (EbA) which uses the Coastal Protection Service naturally provided by marine and coastal ecosystems (Fig. 1A). More generally, EbA is part of the broader Nature-based Solutions (NbS) category, which does not specifically focus on climate change related risk. Scholars use a variety of terms to refer to coastal and climate nature-based adaptation measures, including Nature-based Coastal Defense^3–5^ (NbCD), green and gray-green (with the latter referred to as ‘hybrid’) infrastructure^6,7^, ecological engineering^8,9^, building with nature^7,9,10^, Nature-based Adaptation^11^, and Nature-based Coastal Adaptation^12,13^. As this article focuses on measures aimed at reducing current and/or future coastal risks (i.e. coastal erosion and/or marine flooding and/or compound flooding) driven by climate and non-climate factors using the Coastal Protection Service provided by ecosystems, we refer to Nature-based Coastal Defense (NbCD) hereafter. However, as NbCD is a sub-category of NbS, our thinking is rooted in a broader spectrum of literature.

Hard protection has been extensively used in tropical islands over the past decades, but it has often failed in reducing climate risk^14,15^. Island-specific factors include the poor design and limited resistance of structures to storm events^16–21^, their disproportionately high cost in island contexts^22^, and their complex technicity challenging human skills and capacities locally^23^. More general factors include accelerated urbanization in supposedly protected areas, referred to as the ‘safe development paradox’^24^ or ‘levee effect’^25^; the alteration of risk awareness in these areas^25^; in situ or downstream aggravation of risk by structures^26–29^; and the alteration of ecosystem diversity, complexity and connectivity, which compromises the resilience and potential of ecosystems to adapt to sea-level rise through upward growth and landward migration^30–33^. Such measures that increase climate risk over time and constrain the adaptive capacity of ecosystems are maladaptive^34^. In tropical islands, the failure of hard protection has encouraged the experimentation of NbCD, used separately or in combination with other measures (Fig. 1A). NbCD includes the protection, sustainable management (e.g. pollution control or setback regulations), restoration through direct (e.g. mangrove replanting) and indirect interventions (e.g. hydrological reconnection of ecosystems), and (re)creation of marine and/or coastal ecosystems^35^ (Fig. 1B). Common NbCD measures are reef restoration through coral transplantation^36^; mangrove planting^37^; beach creation or artificial nourishment, with or without vegetation planting^37–39^; and the restoration of the native vegetation of beach and beach-dune systems^40,41^. Hybrid projects combining NbCD with hard protection (e.g. creation of artificial reefs) are also common^6,37,42,43^ (Fig. 1A).

In tropical islands, the increased use of NbCD^44^, and more generally NbS, is supported by the assertion that they allow to overcome the limitations encountered by hard protection. Four main arguments are generally put forward to defend their use. The first one is that ecosystems are effective in providing protection to local communities through wave attenuation and sediment production and trapping^3,4,22,45–51^. Indeed, by reducing wave height by 70%, 36% and 31% respectively, coral reefs, seagrasses and mangroves reduce coastal risks^4^. Second, NbCD is considered inexpensive, due to low initial costs and the self-maintenance of ecosystems, and therefore cost-effective compared to hard protection^4,22,52–54^. Third, NbCD is thought to be easier to implement than hard protection in tropical islands having limited human and technical capacities compared to continental countries^1^. Fourth, NbCD is expected to have multiple co-benefits and no or limited disbenefits, and therefore to be a no-regrets strategy, especially in tropical islands where a disproportionately high number of people receive benefits from ecosystems^22,33,55^. In these settings, NbCD could therefore become a pillar of more sustainable development and even trigger transformational adaptation^56,57^.

However, there is still limited ground-rooted knowledge on (1) the use of NbCD, including the extent to which it is employed, the ecosystems targeted, and the technical measures implemented; (2) the strengths and weaknesses of NbCD compared to other adaptation options; and (3) the internal (i.e. related to NbCD projects per se) and external (generated by external factors) barriers and levers to their implementation and success^3,4,9,41,48,51,58–60^. Four main reasons for this are the high natural variability of ecosystems and their buffering capacity, the limited systematic reporting of risk reduction and adaptation measures, the lack of *ex-ante* and *ex-post* assessments of NbCD, and the variety of stakeholders involved^3,9,61^. To help address these knowledge gaps, this article proposes a systematic assessment of NbCD in five French Overseas Tropical Island Territories (FOTIT). It firstly improves our understanding of NbCD in island contexts and secondly proposes a replicable framework for conducting *ex-post* assessments of NbCD to draw lessons from experiments, and thereby improve the design, implementation, and performance of future projects^62,63^. The results of the assessment allow to re-examine the four arguments put forward to encourage the use of NbCD in tropical islands.

## Study area

**Fig. 2:**
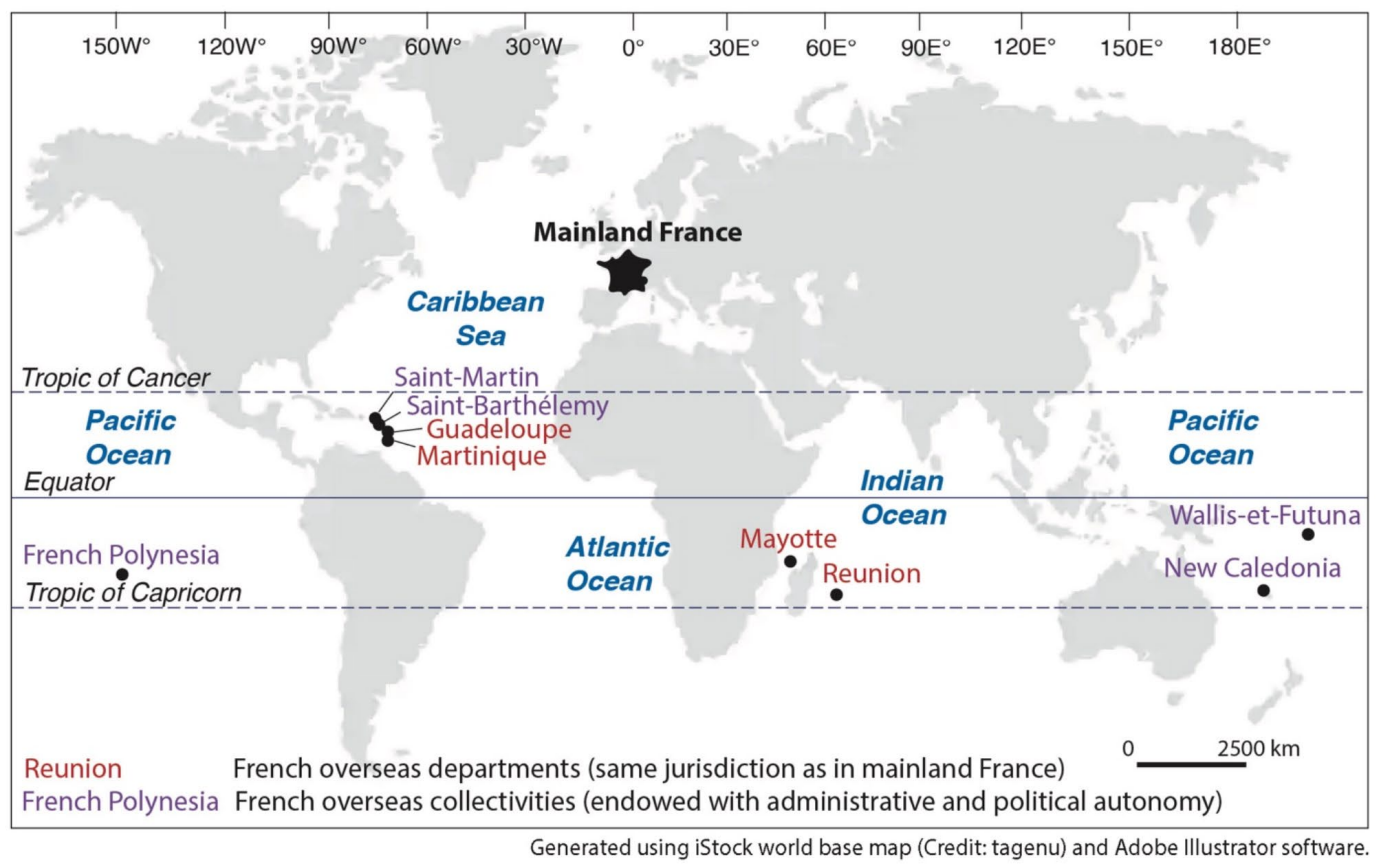
Location of French Overseas Tropical Island Territories (FOTIT). French overseas collectivities ruled by the legislative entity are, with a few exceptions, governed by the same laws and administrative organization as mainland France. The French overseas collectivities ruled by the “legislative speciality” are not automatically governed by national law: the legal status of each collectivity defines its competences and administrative organization which can be quite similar to mainland France (Saint-Martin) or very different (New Caledonia). Saint-Barthélemy and French Polynesia are even granted with autonomy.

This study considers the five French Overseas Tropical Island Territories (FOTIT) in which we were able to identify and assess NbCD projects, including New Caledonia and French Polynesia in the Pacific Ocean; Reunion Island in the Indian Ocean; and Martinique and Guadeloupe in the Caribbean region (Fig. 2). These FOTIT exhibit contrasting physical and human features, with land areas ranging from 1,128 km^2^ to 18,575 km^2^ and including high mountainous and low-lying reef islands, population numbers from 281,118 to 865,506 inhabitants, and diversified economic profiles (SM1). All are to some extent affected by coastal erosion and marine flooding driven by multiple climate and non-climate factors. For example, coastal erosion occurs along respectively 25% and 50% of the coastlines of Guadeloupe and Reunion. The territories having the highest proportion of human assets (buildings, infrastructure, industrial sites) exposed to marine flooding are New Caledonia and Guadeloupe^64^.

## Results

This section presents the mapping of NbCD projects and the results of their assessment, including the analysis of levers and barriers to their success.

### Nature-based coastal defense in French Overseas Tropical Island Territories

**Fig. 3:**
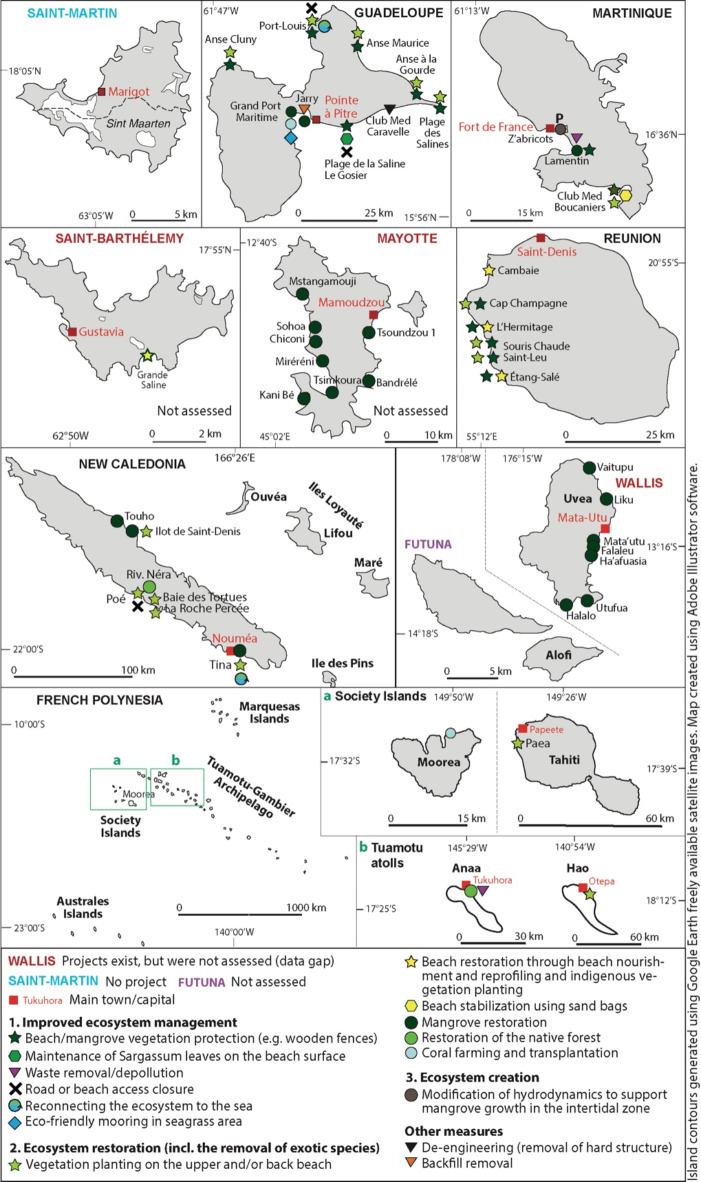
Mapping of Nature-based Coastal Defense projects in French Overseas Tropical Island Territories (FOTIT). Ecosystem protection measures are not included as they do not aim at reducing coastal risks in FOTIT.

We were able to identify and document in full 23 NbCD projects, including 10 in the Caribbean region (7 in Guadeloupe and 3 in Martinique), 9 in the Pacific Ocean (5 in New Caledonia and 4 in French Polynesia) and 4 in Reunion Island (Fig. 3; SM2). Although we also identified projects in Wallis and Futuna (1), Saint-Barthélemy (1) and Mayotte (8), we did not obtain the data needed and therefore could not include them in this study. Six out of the 23 studied projects are multi-site projects. Respectively 11 (47.8%), 8 (34.8%) and 4 (17.4%) projects were deployed in natural or rural areas, peri-urban areas, and urban and/or industrial areas. Concerning the level of human intervention (see Fig. 1 for categories), restoration was widespread, with 13 projects (56.6%) combining the improved management and restoration of ecosystems and 7 projects (30.5%) consisting of restoration actions only, whereas one project (4.3%) consisted of the creation of a new ecosystem. The two remaining projects (4.3% each) respectively combined ecosystem protection, improved management and restoration, and ecosystem improved management, restoration and creation. Nineteen projects (82.6%) targeted one single ecosystem –which is in line with a previous study on Oceania^44^—whereas two projects (8.7%) targeted two ecosystems and two other projects (8.7%) three ecosystems. Among these 29 interventions on ecosystems, 15 (51.7%) concerned beach, dune or beach-dune systems; 8 (27.6%) concerned mangroves; 2 targeted coral reefs (6.9%); and only one intervention (3.4% for each) concerned other ecosystems, namely riparian forest, inland forest (on an atoll), seagrass beds, and salt marshes. Whereas Guadeloupe experienced projects targeting diverse ecosystems, including mangroves, vegetated coastal systems, seagrass, and coral reefs, Reunion Island (where mangrove is absent) only exhibited beach and dune vegetation restoration (Fig. 3).

**Fig. 4:**
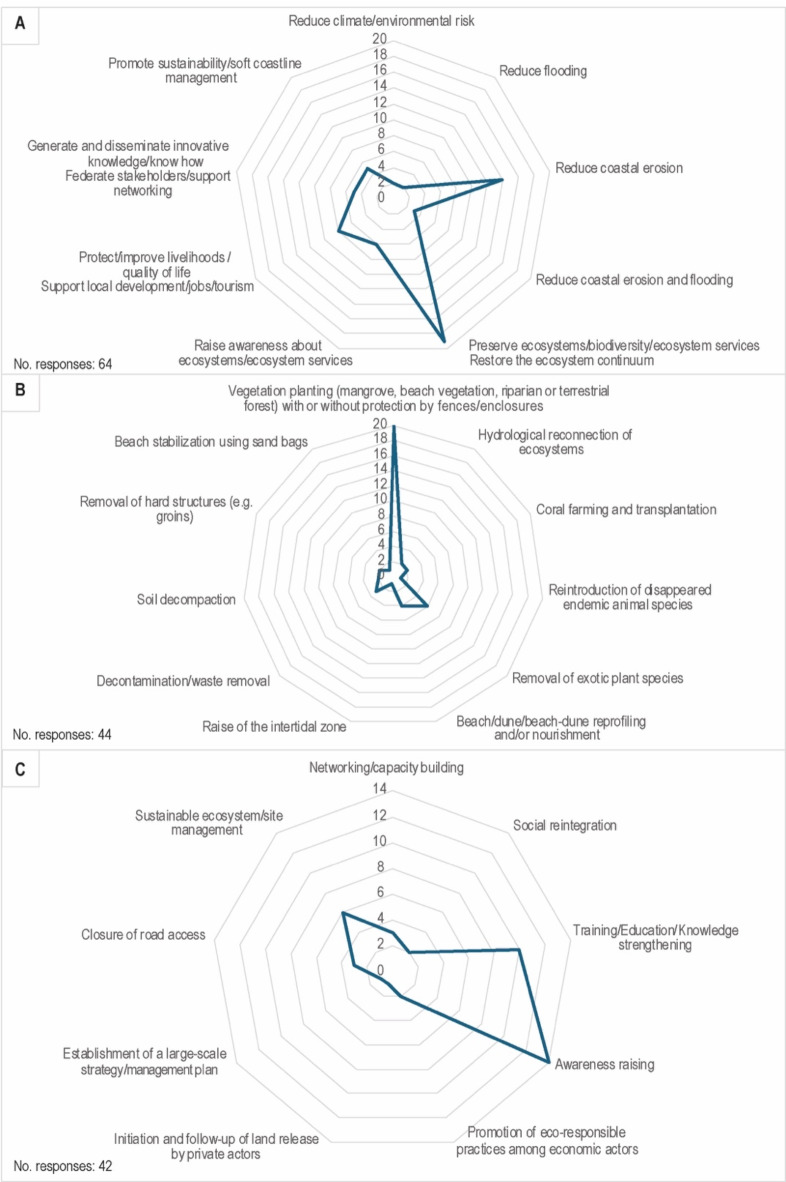
Goals pursued by Nature-based Coastal Defense projects (NbCD), and types of technical and non-technical actions deployed. (**A**) Describes the goals of projects. (**B** and **C**) Respectively detail technical and non-technical actions.

As projects pursued several goals, 64 responses were obtained on this item (Fig. 4A). Their prevailing goal was to reduce risks (21 responses, 32.8%), with 66.7% of projects targeting coastal erosion, 14.3% both coastal erosion and flooding, 9.5% flooding, and 9.5% climate or environmental risk at large. The second goal of projects in order of importance was to preserve ecosystems, biodiversity, or ecosystem services, with 19 responses (29.7%). The third goal was to protect or improve livelihoods or quality of life, including through economic development (8 responses; 12.5%). Three other less represented goals were raising awareness among the civil society (6 responses; 9.4%), generating and disseminating knowledge or know-how (5; 7.8%); and promoting sustainability or soft shoreline management (5; 7.8%).

Implemented actions were both technical (44 actions) and non-technical (42) (Fig. 4B,C). Technical actions fall within 11 types, with vegetation planting prevailing (20 actions; 45.4%), followed by the removal of exotic invasive plant species (6; 13.6%), and beach or beach-dune reprofiling and/or nourishment (4; 9.2%). From a technical perspective, most projects (15; 65.3%) were at an experimental/pilot stage, whereas 6 (26.1%) were at the transfer stage and 1 (4.3%) was partly experimental (beach vegetation restoration) and partly transferred (mangrove restoration). Non-technical actions were dominated by awareness raising (14 projects; 33.3%); training, educational, and knowledge sharing activities (10; 23.8%); and the promotion of sustainable management, for example through waste removal, fire prevention, and eco-friendly anchoring (6; 14.3%). Whereas most projects started in the 2010s (13; 56.5%) and 2020s (9; 39.1%), 9 projects were preceded by previous projects deployed at the same location. On the north-east coast of New Caledonia, mangrove restoration was first implemented in the 1980s by Kanak tribes. In Reunion and Guadeloupe, beach vegetation restoration was first experimented in the late 1990s-early 2000s. Since then, continuous action was taken to strengthen these ecosystems at these locations. The duration of projects varied greatly, with 6 projects lasting from 1 to 3 years, 2 projects from 3 to 6 years, 2 projects from 6 to 9 years, and 10 projects being run over the long-term (> 10 years or guaranteed in the future because falling within the mission of a public holder).

Most projects were led by public actors (11 projects; 47.8%) and associations (8; 34.9%) (SM2). Among the former, the local authorities prevailed (5; 21.7%) over national bodies such as the National Forestry Office and the Coastal Conservatory (4; 17.4%). In addition, two projects involved both local and national public actors. Other project holders included tourism companies, the Great Harbor of Guadeloupe, and a partnership between an association and the national government.

Most projects relied on diverse funding sources, including funds dedicated to NbS/EbA from the International Union for Nature Conservation (IUNC); the European BEST and LIFE initiatives and FEDER; the Pacific KIWA initiative (https://kiwainitiative.org/en/about-kiwa-initiative - mapzoneactions); Conservation International; the Pacific Regional Environment Program (SPREP); and the French development agency (AFD) (SM2). In addition, national bodies involved in coastal conservation and management (e.g. National Forestry Office, Coastal Conservatory, French Office for Biodiversity, Ministry of Ecological Transition, locally based environmental divisions) and municipalities funded some projects. Private funding was only involved in three projects, led by a tourism company and the Grand Port of Guadeloupe. Interestingly, in New Caledonia and French Polynesia, volunteering and mutual aid were key contributors in three projects. The total cost of most projects was difficult to evaluate, as multiple funding sources were involved over different time periods and some costs (e.g. salaries of permanent staff) not estimated. In some cases (e.g. tribes), evaluating the total cost of projects simply did not make sense for project holders.

### Assessment of nature-based coastal defense projects

**Fig. 5:**
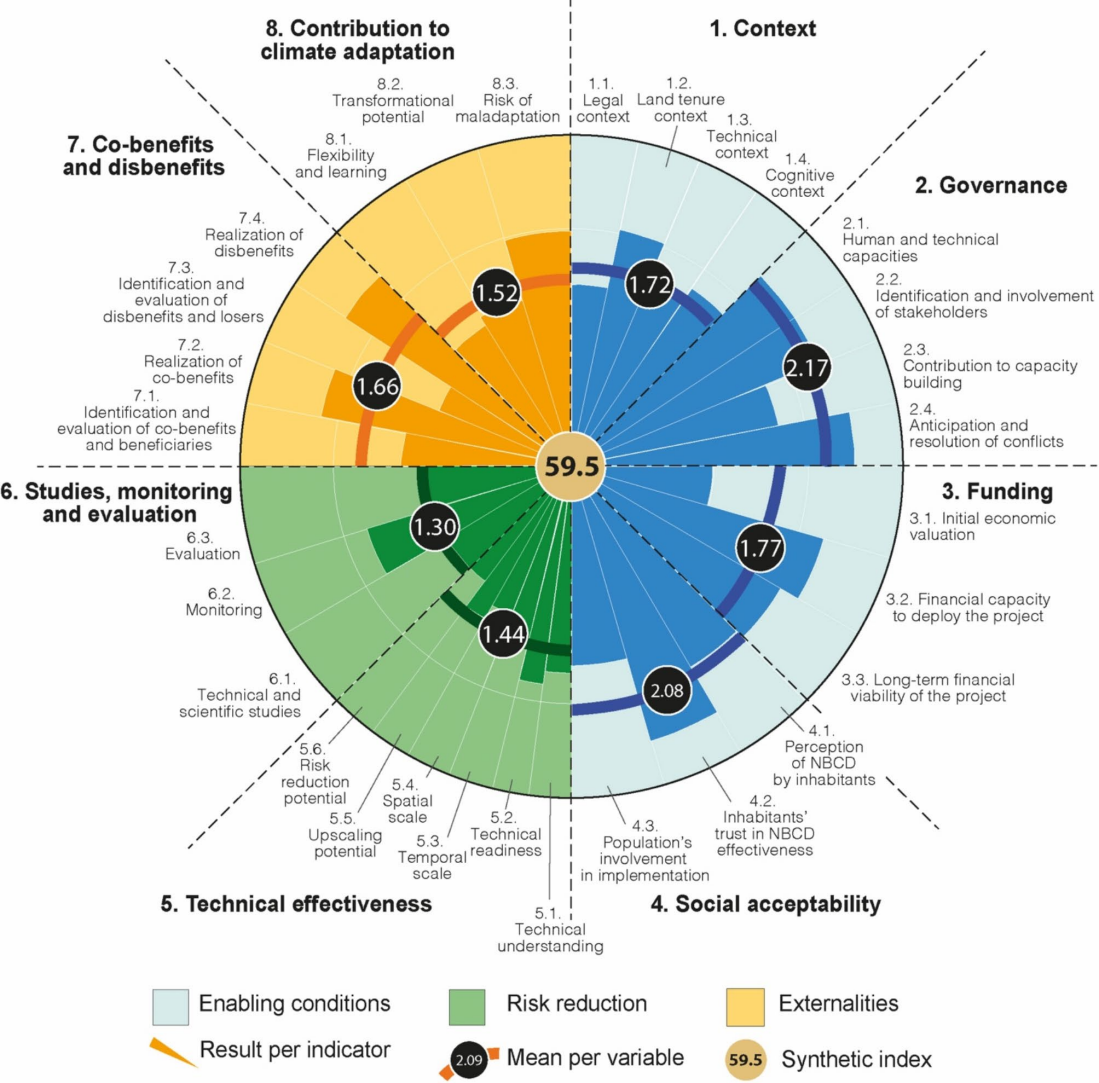
Synthesis of the results obtained for the 23 Nature-based Coastal Defense projects assessed in this study. See SM7 for the radar chart of each project. The assessment framework includes three domains (Enabling conditions, Risk reduction, and Externalities) encompassing 8 variables numbered from 1 to 8 in this figure, and 30 indicators (e.g. 1.1, 1.2, etc.).

At project level, the assessment revealed marked contrasts, with performance indices ranging from 39.4% (project No.20 in SM2) to 77.2% (No.13) for a mean of 59.5% (Fig. 5; SM3 and SM4). The three best-performing projects (> 70%) were carried out by an association restoring turtle nesting beaches on the west coast of Reunion Island since the 1990s (No.13; 77.2%); a Kanak tribe applying mangrove restoration methods developed by their elders at Poindimié, New Caledonia (No.5; 73.9%); and the municipality of Lamentin, Fort-de-France, Martinique, which initiated back-mangrove restoration in polluted industrial areas in the 2010s following cyclone devastation (No. 15; 71.1%). Projects with the lowest scores (< 50%) were led by a tourism company in Martinique (No.16; 44.3%) and Guadeloupe (No.20; 39.4%), and by a local association on Anaa Atoll, French Polynesia (No.8; 45.4%). These projects exhibit weak scores for many variables, including Context, Governance, Technical Effectiveness, and Studies, Monitoring and Evaluation. The forest restoration project deployed on Anaa Atoll, French Polynesia, faced persistent difficulties of all kinds, not only material and due to a lack of experience in project management and technical skills, but also related to governance and social acceptability due to an acute conflict with the municipality. Between these two extremities, most projects have scores ranging between 50 and 65% (SM3).

At the level of variables, numbered from 1 to 8 in Fig. 5, the results emphasize strengths and weaknesses (SM3 and SM4). In general, projects exhibit medium (1 < x < = 2 on the 0–3 scale used) to high (> 2) scores on enabling conditions, including governance (mean: 2.17/3; Fig. 5) and social acceptability (2.08/3; Fig. 5). These findings firstly reflect the ability of project holders and partners to design, initiate and lead NbCD projects, involve stakeholders in their deployment and daily management, and resolve conflicts where they arose. A structured, mobilized, and efficient community of practice having recognized skills exists in FOTIT, mainly composed of institutional actors in the Caribbean region and in Reunion Island, and of local associations in Pacific Ocean collectivities. However, this community of practice mostly operates in a closed circuit at the level of each FOTIT, with limited exchange with neighboring countries, other FOTIT and even, in some cases, other actors of the same territory.

The findings secondly indicate the population’s general support to NbCD. Depending on FOTIT, 78.1–93.3% of the surveyed population were in favor of such solutions for reducing risks (SM4). In all FOTIT, most interviewees expressed a rather high to very high confidence in the effectiveness of NbCD, against a small proportion who declared having limited confidence in this option. The highest levels of confidence were obtained in the Pacific, especially in the Kanak communities of New Caledonia implementing mangrove restoration practices. In these settings, this solution was initiated in the 1980s after two decades of deforestation that had led to increased coastal erosion and storm-driven damage. The strong confidence of these communities in mangrove restoration is rooted in the direct observation of the risk reduction benefits provided, including beach replenishment and decreased wave attack. In French Polynesia, depending on the location, 43.8–53.8% of interviewees declared having high to very high confidence in NbCD; 21.9–36.5% having a rather high confidence in this option; 7.7% (Moorea) to 28.1% (Hao Atoll) having low confidence in NbCD; and 1.9–8.0% having no opinion (SM4). Likewise, in Martinique and Guadeloupe, 76% of interviewees declared having rather high to very confidence in NbCD, against 73.3% in Reunion Island. However, the concrete involvement of the population in projects remained limited on average (1.65; Fig. 5), ranging from nil for three projects (Nos. 16, 19 and 20 in SM2) to very high for five projects (Nos. 2, 5, 13, 15, 18). The former include the two projects led by hotel companies that do not seek to involve the population, whereas the latter include projects specifically aimed at raising public awareness that were successful in doing so, as well as projects based mainly on voluntary work (e.g. in Kanak communities).

Concerning enabling conditions, scores were lower for the Context (1.72/3) and Funding (1.77/3) variables (Fig. 5; SM3 and SM4). Favorable contextual factors include public land tenure, which acts as a major lever to NbCD under the impetus of the Coastal Conservatory and National Forestry Office in the Caribbean region and in Reunion Island. This explains higher scores for land tenure in these FOTIT compared to Pacific territories, where these public actors are absent and land tenure is much more conflictual, a legacy of the dispossession operated during the colonial period and still unresolved. In general, the cognitive context also acted as a strength, with most projects benefitting from appropriate knowledge on and understanding of the protection service provided by ecosystems and the conditions under which this service can be delivered. This knowledge was mainly derived from technical and scientific studies in the Caribbean and Reunion Island, and from both local knowledge provided by individuals (i.e. experts) and groups (associations) and indigenous knowledge held by customary authorities and elders in French Polynesia and New Caledonia. However, stakeholders pointed out knowledge gaps on swamp forest and on complex land-to-sea hydrological connections as a limit to effective action at some locations. As far as funding was concerned, most interviewees said they had sufficient funds to carry out their project and were confident in their ability to sustain it beyond the limits of ongoing multi-year funding. The reasons put forward were that ecosystem restoration is their main mission (i.e. environmental associations, and local-to-national institutional actors in charge of ecosystem protection and management); ecosystems are vital to them, acting as food providers and a support to cultural heritage (Kanak tribes); environmental compensation measures will continue to apply (Grand Port of Guadeloupe). However, all project leads confessed that calls for projects were not well-adapted to nature-based actions requiring a long-term involvement. Regulations had a neutral impact on some projects that they did not facilitate nor constrained. On the other hand, they acted as a facilitator for some projects (e.g. the national turtle protection plan facilitating the implementation of beach vegetation restoration projects) and as a barrier for others (e.g. constraining rules for intervention on protected plant or animal species; heavy public procurement or environmental impact assessment procedures). In most cases, project holders complained about the cumbersome nature of regulatory and administrative procedures, including those required to obtain funding, and their negative impacts on project management, such as longer implementation times, heavy reporting, and the need to have dedicated staff for project engineering.

Medium scores were obtained for externalities –which cover the positive and negative impacts of projects beyond local risk reduction– with mean scores of respectively 1.66/3 for co-benefits and disbenefits and 1.72/3 for project contribution to adaptation (Fig. 5; SM3 and SM4). Overall, this study confirms that NbCD generates limited disbenefits and losers and is not maladaptive^65,66^. The only maladaptive effect identified in our sample was the continued planting of an introduced tree species (*Casuarina equisetifolia*) aggravating coastal erosion in association with native species to encourage public appropriation of a beach restoration project (No.11, Reunion Island; SM2, SM4). The project holder justified this choice by the public’s attachment to the landscape and shade associated with this tree species and considered that its eradication could have condemned the project. This choice, which is debatable in a territory where other stakeholders have engaged in the systematic removal of invasive plant species, shows that social acceptability is recognized as a major factor in project success. In line with a recent review^59^, our results confirm that the benefits and disbenefits generated, and the nature and number of beneficiaries and losers, were not precisely assessed. Project holders confessed that their difficulty in demonstrating co-benefits even reduced the support they received from elected representatives. Last, because they were mostly experimental, carried out on a very local scale, and not integrated into a broader adaptation strategy (i.e. non-existent or emerging, depending on FOTIT), most projects were incremental and had a limited contribution to adaptation at the territory scale. Flexibility, conceived as an implementation principle, was constrained by the rigidity of the rules governing funding arrangements (e.g. impossibility of redirecting funds to meet actual needs).

Last and importantly, NbCD projects have relatively low scores on risk reduction variables, with mean scores of 1.44/3 and 1.30/3 on technical effectiveness, and on studies, monitoring and evaluation, respectively (Fig. 5; SM3 and SM4). This can be seen as a paradox, as most projects primarily aimed at reducing coastal risks. However, their risk reduction potential was greatly reduced by their experimental nature and the small spatial scale at which they were implemented (a few tens to hundred meters of coastline, a few dozen square meters or decimeters). Because most projects did not include the reduction of climate change risk as an objective, they did not consider long timeframes. As current risks were not systematically assessed and future risks not assessed, technical actions were not calibrated accordingly, making the risk reduction potential of most projects highly uncertain under climate change. In addition, most projects mainly relied on ecological studies and were conceived more as ecological engineering projects than complex social projects. In these projects, the little attention given to enablers, especially governance and social acceptability, reduced their risk reduction potential by generating barriers to implementation and success. Yet, most projects benefitted from the good understanding that project leads had of the geomorphic context and of how the technical actions undertaken can reduce risk; from their technical readiness to implement technical actions; and from monitoring activities. But the latter generally focused on the success of ecosystem restoration, using indicators such as the survival and growth rate of reintroduced plants, disregarding indicators aimed at evaluating the risk reduction benefit provided by ecosystems.

## Discussion

These grounded results allow us to reexamine the four arguments presented in the introduction and advocating for the technical effectiveness, low cost, ease to implement, and no-regrets character of NbCD.

### Does nature-based coastal defense reduce risk?

Four main reasons prevent us from providing a definitive answer to this question. First, some projects are still at an experimental stage at which they cannot reduce risk^57^. Second, because most projects were recently deployed, restored ecosystems are not yet mature, and therefore not yet able to provide the expected risk reduction benefits. Third, where restored ecosystems are mature (e.g. mangroves in New Caledonia), they have not yet been affected by intense climate events, making it possible to verify their ability to withstand such events and to provide effective protection to coastal communities. Fourth, the effectiveness of the protection provided by ecosystems will only be known in the future if the required long-term monitoring aimed at evaluating their technical effectiveness –which is currently lacking– is put in place^4^.

Although these four factors limit our ability to answer the question of NbCD effectiveness, several points can be made. At this early stage of implementation, the lack of experience of projects’ holders and of appropriate measurement of effectiveness constrain the wider acceptance as well as the upscaling of NbCD^3,31^. This reduces the potential for NbCD to be an efficient vehicle for transformative change by supporting transitions towards sustainability and transformational adaptation^67^.

We were able to identify internal (i.e. related to NbCD projects *per* se) and external (i.e. related to external drivers) factors that are likely to respectively reinforce (i.e. act as levers) and reduce (i.e. act as barriers) NbCD projects’ effectiveness when they reach maturity. Levers include the solid experience and know-how acquired by Reunion and Guadeloupe stakeholders on beach and dune vegetation restoration, based upon the use of a variety of restoration techniques adapted to context specificities, including with or without beach or dune nourishment and reprofiling, with or without protection enclosures, through vegetation natural regrowth or replanting. Most projects combined restoration techniques with improved beach and dune management, e.g. the closure of access roads and promotion of soft mobility and reversible equipment, user guidance and awareness-raising. Likewise, in Martinique and New Caledonia, project holders have mastered mangrove restoration techniques that have proved their worth in different territorial contexts, including urban (Nouméa, New Caledonia), industrial (e.g. Fort-de-France, Martinique) and rural (e.g. tribal Kanak villages, north-east coast, New Caledonia). This know-how has been transferred to new sites through multi-site projects and the spatial extension of experimental sites, and to new operators such as tourism companies. In Reunion Island, beach and beach-dune vegetation restoration has even reached the ‘landscape scale’^6,68^, as it was recently carried out at the scale of entire sediment cells and is now in the process of being extended to the entire west coast. This successful experience could serve as an example for NbCD upscaling.

However, two external barriers reduce the technical effectiveness of NbCD projects. The first one is the alteration of marine environmental conditions in peri urban and urban-industrial areas, which condemned reef restoration operations to failure. This barrier, which was pointed out by studies conducted in the Caribbean region^51^, underlines the need to first tackle the root causes of ecosystem degradation (i.e. pollution) before carrying out restoration^62,63,69,70^. Such a strategy was deployed in beach restoration projects focusing on eliminating invasive plant species before replanting native species in Reunion Island. This example could thus inspire future restoration projects. The second external barrier to technical effectiveness relates to the lack of acculturation of local actors to the climate change challenge^71^, which encourages the maintenance of incremental strategies where transformative action is needed. In Caribbean territories, a good example is the transfer of beach restoration actions to new sites. While this strategy gradually extends restored perimeters to the limits of public land, it however does not allow for the scale-up required to optimize risk reduction benefits now and in the future. Although they are increasing in number and in spatial coverage, Caribbean NbCD projects are still mainly confined to public land and scattered, rather than being integrated into a broader transformational strategy applying to coastal areas at large^9,72^. Rolling out the National climate change adaptation plan (PNACC) to each of the FOTIT could help moving from the current incremental approach to a transformational one prioritizing NbCD by providing the required space to ecosystems where NbCD is relevant, while also reinforcing the risk reduction potential of future projects through the integration of future risk levels in their calibration. Integrating NbCD into a broader and longer-term strategy would also allow to acculturate stakeholders to increasing climate change pressures that could, depending on the climate scenario that will unfold, lead to the failure of actions that would not have been calibrated accordingly^31^. Embedding NbCD into the climate adaptation policy would finally help to operationalize the latter, while in turn generating the “substantive” (stakeholders’ knowledge informing and improving planning), “instrumental” (stakeholders’ understanding, acceptance of, and support to the process) and “normative” (increased legitimacy of the process) governance benefits^56^ that are required to break political-institutional path-dependencies and support the transition to transformational adaptation^60^.

### Is nature-based coastal defense inexpensive and more cost-effective than other options?

In line with previous studies^4,60,73^, our results highlight the lack of economic evaluation of NbCD projects and beyond, their failure to assign a monetary value to the full range of ecosystem contributions to human well-being. Moreover, trying to meet this objective simply did not make sense for Kanak communities, whose way of life is mainly based on subsistence activities. In most cases, the economic evaluation of projects only included an estimation of their cost, which was a *sine qua non* condition for obtaining funding. In general, the total cost of projects, including for example the salaries of permanent staff or volunteering work, remained unassessed. In addition, no project included a cost-effectiveness analysis or a compared analysis of the cost of NbCD and other options.

In line with previous findings^60^, most project holders stressed the inadequacy of existing financial tools mainly consisting of multiannual calls for projects and of their terms and conditions to support ambitious and long-term NbCD. In most cases, they confessed that they had under-evaluated the cost of their project, due to the underestimation of some costs (personnel, studies and monitoring) and the unpredictability of certain expenses driven by weather conditions and the success rate of plantations (e.g. cost of watering seedlings or replacing dead plants). Overall, their experience was that NbCD is more costly than expected, and that large-scale projects could not be carried out with current human capacities because of the high staffing requirements for their maintenance, which aligns with previous analyses^41^. This would act as a major barrier to the scaling up of vegetation restoration. In addition, even in the case of projects supported by European funding, holders acknowledged having limited project engineering capacity to meet all their obligations, from responding to calls for projects to reporting on implementation right through to final evaluation. Last, most project holders mentioned the difficulty of securing long-term financing, regretting the discontinuous nature of their actions. Even key public actors mentioned the alternation of project phases, during which actions were carried out, and inter-project phases, during which they were forced to reduce actions to a minimum. In contrast, projects led by well-established local associations and supported by voluntary work were much less affected by these constraints. This underscores the crucial role of empowering local communities, even non-indigenous, if NbCD was to be extended to support transformational climate adaptation where relevant^68,74^.

### Is nature-based coastal defense easy to implement?

In island contexts, NbCD has two advantages over hard protection: it does not require costly imports of materials (as hard protection does in atoll contexts) and it can rely on locally available valued ecosystems and local and potentially indigenous knowledge^52^. However, these advantages are not enough to make NbCD easy to implement. Although the popularity of NbCD and existence of a mobilized, structured, multi-profile, and experienced community of actors facilitated project implementation in studied islands, project leads identified key external barriers to implementation. In addition to those related to knowledge gaps and the limited human, technical and financial capacities of FOTIT, they noted the lack of strong political support to NbCD, as well as the limited involvement of the population in contexts where there is a strong disconnect between lifestyles and ecosystems. This situation for example occurs in urban and peri urban areas of Caribbean islands and Reunion Island. This disconnect and more generally the lack of understanding of climate change related challenges by the civil society and by decision-makers explain public opposition (i.e. vandalism, theft of equipment, and sabotage of actions) to some projects implying major changes in beach accessibility conditions and uses and a strong political-institutional path-dependency to hard protection in most territories. These insights are in line with previous studies proposing approaches aimed at overcoming governance-related barriers and promoting the empowerment of local communities and the better integration of their knowledge and sense of place, and of the co-constitutive thinking of Indigenous communities (i.e. non-dichotomous vision of the relationship between humans and nature) to ensure the successful design and implementation of NbCD^63,67,75,76^.

### Is nature-based coastal defense necessarily a no-regrets strategy?

Answering this question based on our sample is made difficult, first, by the lack of detailed evaluation of the co-benefits and disbenefits generated by projects, and second, by the multi-objective nature of projects which reduces the potential for co-benefits. Project holders unanimously defended the no-regrets nature of NbCD in view of its many potential benefits, in line with the arguments put forward by some scholars^77^. However, the absence of a long-term strategy could be highly detrimental to NbCD, which is supposed to ‘*have low initial appeal (…) but increasing social and ecological benefits over time*’^52^.

## Conclusions

Based on grounded results collected in five tropical island territories, this paper discusses the generally accepted, yet poorly demonstrated, ideas about the added-value of Nature-based Coastal Defense (NbCD) for reducing coastal risks and adapting to climate change, and beyond, supporting the transition to transformational adaptation in island territories. The findings nuance the positive framing of NbCD claiming that it constitutes a readily employable and effective, inexpensive and cost-effective, easy to implement, and no-regrets adaptation solution. In our sample, few projects currently have a high potential for climate adaptation, as some critical steps such as upscaling actions at risk reduction-relevant scale, ensuring long-term funding for their perpetuation, calibrating technical actions according to future climate risk, monitoring the risk reduction benefits generated, and enrolling and involving over the long run concerned stakeholders including elected representatives and the civil society, have not been reached yet. This shows that NbCD still faces major limitations and challenges to meet expectations and become a robust adaptation solution. Some of these challenges (e.g. scaling-up action or ensuring long term funding) are not NbCD-specific and constitute more general coastal adaptation challenges also applying to other adaptation options such as relocation and (is)land raising. In contrast, some limitations and challenges raised in this study are specific to NbCD (e.g. ensuring suitable environmental conditions in restored areas or establishing protocols to measure the risk reduction benefits provided by ecosystems).

The findings of this study are currently used by project holders to strengthen their projects, especially through the design of monitoring protocols that will allow to evaluate risk reduction benefits. In addition, experience sharing promoted through the organization of on-site final workshops in each FOTIT helped project holders to identify ways to overcome some internal barriers to NbCD, especially in four areas: (1) reinforcing technical effectiveness through the replication of successful technical actions concerning, for example, plant reintroduction, maintenance and watering; (2) ensuring long-term funding through increased awareness of funding sources and the strengthening of project engineering capacities; (3) meeting NbCD maintenance requirements through partnerships with local associations and reinsertion contracts; and (4) reducing the negative impact of cumbersome administrative and regulatory procedures (e.g. delays, discouragement) on project implementation through the anticipation of these constraints, and more supportive collaboration with relevant national bodies.

Our assessment method allows to measure the multi-dimensional status of NbCD, gathering multiple perspectives (scientific, technical, practitioner-related, societal) and bringing grounded evidence to ongoing theoretical debates about the added value of NbCD for long-term coastal adaptation. This methodology, which was designed to conduct *ex-post* assessments of NbCD, was recently used as an *ex-ante* assessment method by the French Ministry of Ecological Transition to select the winning projects in the second national call for NbS projects (2024–2029). This highlights its usefulness to serve both as an *ex-ante* and an *ex-post* assessment tool. In addition, NbCD project holders and partners of study territories suggested to use this assessment protocol as a regular monitoring tool, that is, to monitor the progress of their projects over time and to evaluate the new projects implemented in their territories. Lastly, because it is based on generic variables and indicators, our assessment protocol could be used to also assess other coastal adaptation options. This would make it possible to compare the effectiveness of various adaptation measures in each territory or country and to distinguish between generic and NbCD-specific levers and barriers to coastal adaptation.

## Methods

### General approach at the science-practice interface

This study was carried out as part of the ADAPTOM (2022–2025; https://adaptom.recherche.univ-lr.fr/) and FUTURISKS (2022–2028) https://futurisks.recherche.univ-lr.fr/en/projet-en/) research projects, designed to meet the demand expressed by the FOTIT’s actors for feedback on NbCD projects (*What lessons can be learnt from past and ongoing projects?*), scientific support to successfully deploy them (*What are the conditions for success?*), and experience sharing (*What capacity-building activities can strengthen local skills and promote knowledge and know-how transfer?*). The working method involved close collaboration between researchers and actors, facilitated by the creation of a steering committee composed of eight actors representing the FOTIT NbCD community. The projects also involved academics (‘researchers’ hereafter) from mainland France and FOTIT, as well as non-academics (‘actors’) working in FOTIT’s institutions, associations and organizations (e.g. museums or botanical gardens), with expertise in marine and plant ecology; coastal geomorphology; environmental, planning and risk law; human geography; climate risk and climate adaptation. The size and composition of this team varied over time to meet needs. The projects involved institutional actors from municipalities and communities of municipalities, departmental and regional services, and statal and para-statal divisions (esp. Environment division, National Office for Biodiversity, National Forestry Office, Coastal Conservatory, Agency for the Environment and Energy Management), as well as representatives of local associations, NGOs, and private companies (e.g. tourism and engineering). The boundary between researchers and actors was permeable, with some participants being both researchers and actors.

**Fig. 6:**
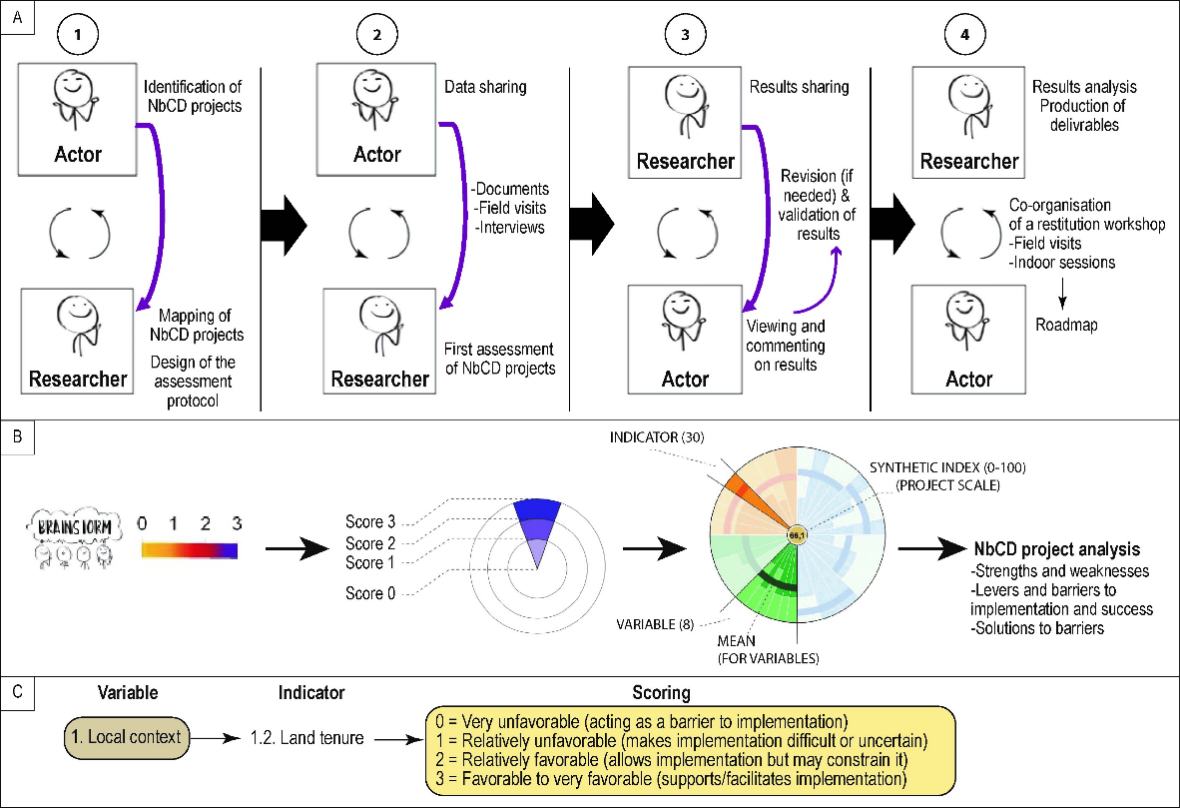
Collaborative protocol aimed at assessing Nature-based Coastal Defense projects. (**A**) Describes the four stages of the researcher-actor collaboration, emphasizing the key role of stakeholders at the various steps of the assessment. (**B**) Summarizes the scoring system used to assess projects, from the attribution of scores to the drafting of the final radar chart and derived analysis. (**C**) Uses an example to explain the scoring of the 30 indicators used in this assessment.

The co-evaluation of NbCD projects was carried out in four stages (Fig. 6A). The first step included the identification of projects by local actors and their mapping by researchers, who also designed and submitted the assessment protocol to actors for discussion. The second stage involved data collection by researchers among projects’ leads and partners, as well as an initial assessment of projects by researchers. The third stage was dedicated to sharing the results of the assessment with the actors. In cases where actors’ feedback highlighted points for discussion, a videoconference was organized to refine the results, which were finally validated by both parties. The last stage consisted in the analysis and valorization of the results through non-academic deliverables and the co-organization of a restitution workshop in each FOTIT involving representatives from other FOTIT. The duration of the workshop varied according to the number of NbCD projects, and the program included four activities: knowledge-building sessions prepared by researchers, on-site and indoor presentations of NbCD projects by project leads and partners, detailed presentations by researchers and discussion by the group of the results of the assessment, and a final session dedicated to drawing up a roadmap for future activities. The number of participants was limited to 26 for field visits to create optimum conditions, but it was not limited for indoor activities (maximum number of participants: 75). Participants were chosen based on an expression of interest, and with the aim of enabling a wide variety of profiles to take part to workshops. After each workshop, participants evaluated the workshop and the deliverables. This allowed the scientific team to learn lessons from one workshop to another, and to respond as effectively as possible to actors’ needs.

### Assessing nature-based coastal defense projects

The analysis of NbCD projects included their detailed description and evaluation.

*Step 1*: Mapping nature-based coastal defense projects.

In line with previous studies^37,78^, key descriptors were used to characterize projects: project number (e.g. No.1); ocean basin (e.g. Pacific 1); project full name; location (island, municipality); mono- or multi-site project; urban, rural, peri-urban context; targeted ecosystem; main goal(s) pursued; technical and non-technical (e.g. awareness raising) action(s) implemented; degree of technical mastering (experimental, mastered, transferred); dates of implementation and of first action, prior to the project, if any; duration; project holder; funding source(s), and total cost.

*Step 2*: Designing an ex-post assessment protocol.

*Ex-ante* assessments of NbS made considerable progress, using physical modelling, and cost-benefit and multi-criteria analyses^59^. Yet, these assessments still face challenges in evaluating and incorporating intangible benefits of NbS, stakeholders’ preferences and mobilization, as well as trade-offs and conflicts generated^59,60^. In addition to *ex-ante* assessments, *ex-post* evaluations are needed to learn from experience and promote more appropriate options and implementation procedures in the future^4,42,79,80^. To help address this need, we propose an *ex-post* assessment protocol which builds on previous studies, including the IUCN global standard for NbS and uses a Structured Expert Judgment (SEJ) method based upon a scoring system^81,82^.

**Fig. 7:**
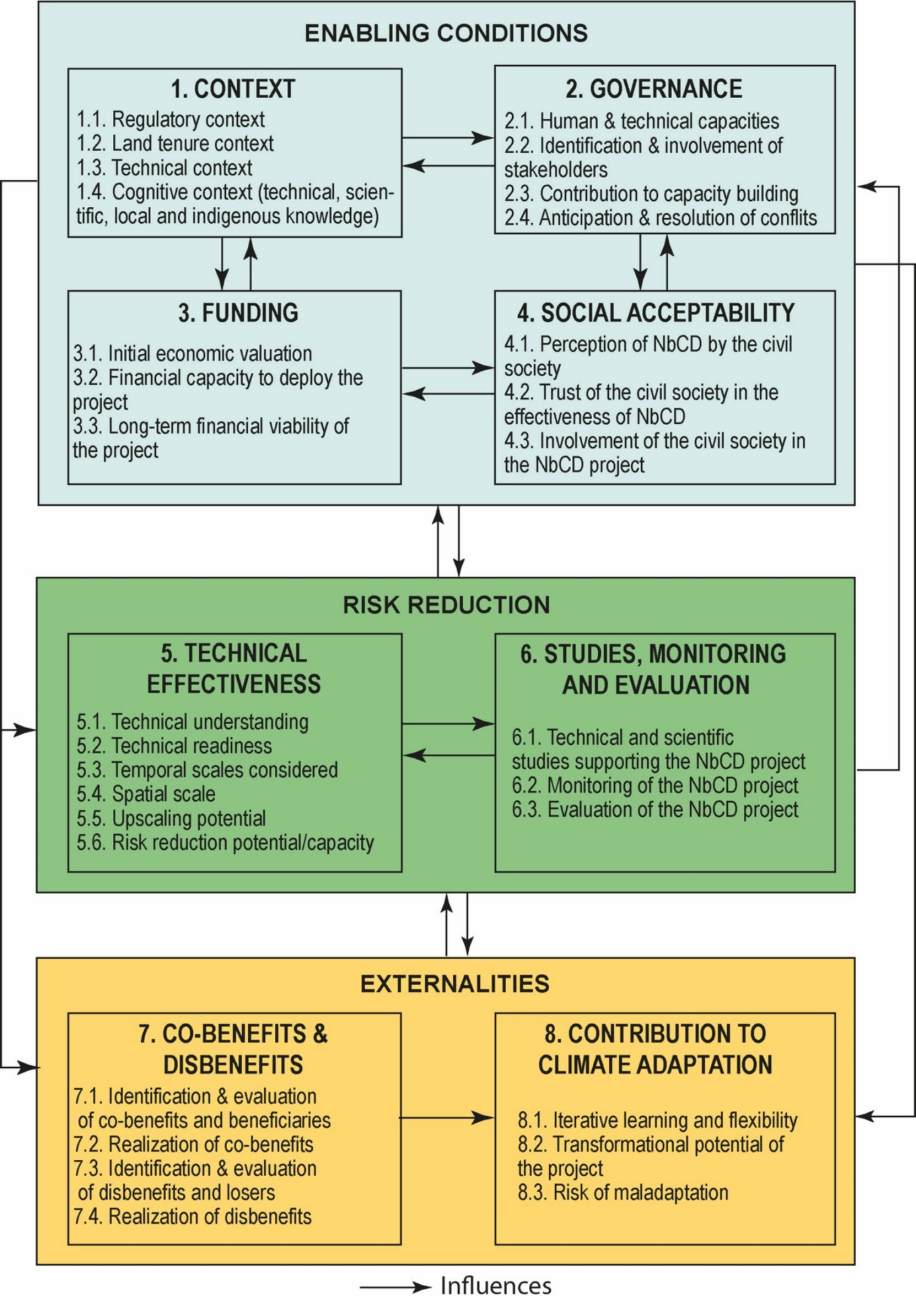
Structure of the assessment protocol used to analyze Nature-based Coastal Defense projects. It considers three main domains (enabling conditions, risk reduction, and externalities), eight variables (numbered from 1 to 8 in the chart) and 30 indicators (e.g. 8.1).

This protocol considers three main domains encompassing eight variables and thirty indicators (Fig. 7). The three domains considered are enabling conditions, risk reduction, and externalities. At the variable and indicator levels, the assessment responds to a guiding question. Hereafter, we briefly describe the variables and indicators used (see SM6 for details). Enabling conditions are the conditions allowing and facilitating the deployment and success of the project, and they include four variables. Recognizing that risk reduction and adaptation are context-specific, variable 1 relates to the local and national context, as national laws and policies apply to a certain extent in FOTIT. It was assessed using four indicators addressing respectively the regulatory, land tenure, technical, and cognitive contexts. Variable 2 refers to governance, assessed through four indicators: the human and technical capacities of the holder and its partners to implement the project^41,72^; their capacity to involve concerned stakeholders^59^; the project’s contribution to capacity building; and the ability of the project’s holder and partners to anticipate and resolve conflicts^2^. Variable 3 relates to funding which is captured through three indicators, namely the realization of an initial economic evaluation, the financial capacity to deploy the project, and its long-term viability^41,60,66^. Variable 4 covers social acceptability, defined as the capacity of a community to support the implementation of a measure^31^. Initially, our aim was to assess the perception of NbCD projects by inhabitants, their trust in its risk reduction potential, and their implication in its deployment and maintenance^80,83^, using interviews with professionals, and a population survey (wherever individual questionnaires were welcome) or focus groups (in tribal areas, where they had the preference of customary authorities and associations). But our test survey revealed that most inhabitants were unaware of projects and therefore unable to answer our questions. To maintain social acceptability as a variable in this assessment, we adjusted the questions, asking respondents their opinion on the various types of risk reduction measures including NbCD, and if they trusted NbCD to reduce effectively risks. Additionally, the involvement of the civil society in the deployment and maintenance of projects was analyzed based on the factual data provided by project holders.

The second domain considered in this assessment is risk reduction, which refers to the capacity of NbCD projects to reduce coastal erosion and/or marine flooding and/or compound flooding. We assessed risk reduction using two variables, namely the technical effectiveness of projects^35,39,84^ (variable 5), and the extent to which they are supported by technical and scientific studies, monitoring activities, and evaluation^59^ (variable 6). Technical effectiveness was captured using six indicators: the technical understanding of risk and how the project can reduce it by project holders and partners; their technical readiness to implement actions; the temporal scales considered, especially the lead time until full effectiveness and the duration of risk reduction benefits; the spatial scale considered, ideally landscape or system scale^68^, e.g. sediment cell; the upscaling potential of the project; its risk reduction capacity or potential.

The third and last domain refers to the positive and negative externalities generated by the NbCD project, including its co-benefits and disbenefits^22,84^ (variable 7), and its overall contribution to climate adaptation (variable 8). Variable 7 comprises four indicators, including the identification and evaluation of co-benefits and beneficiaries, the realization of co-benefits, the identification and evaluation of disbenefits and losers, and the realization of disbenefits. Variable 8 comprises three indicators, namely the uptake of iterative learning and flexibility in project implementation, its transformational potential, and the risk of maladaptation associated with it.

Variables and indicators were documented using the analysis of project-related documents, observation data collected during site visits with project holders and partners, semi-structured interviews with project holders and partners (2 to 4 recorded interviews per project; SM7) and with local professionals, and a population survey^85,86^ (target: 50 local site users). In conflictual contexts, we faced difficulties to generate the social acceptability indicators. The latter were documented as “no data” and not included in the results.

### Building of databases

The data collected was integrated into three distinct databases. Database 1 gathers all the data that was collected for each project, using the structure of the interview guide (SM7). This database served as the main data source during the analysis. Database 2 is an excel file that follows the domain/variable/indicator structure of the assessment protocol and includes for each project the scores attributed to each indicator (see next section) and their justification (SM4). Built from database 1, database 3 gathers for each project qualitative information on the levers and barriers to its implementation and success, including relevant quotes extracted from interviews with project holders and partners.

### Project evaluation and analysis

For each project, the 30 indicators were assessed using a scoring system ranging from 0 (lower score) to 2 or 3 (higher score), depending on the indicator (Fig. 6B,C). A methodological notice was developed to detail the scoring system and thereby allow assessors to have the same understanding of scores. The attribution and justification of scores was first performed separately by the two researchers who carried out data collection and were involved in the project throughout its duration. Scores were compared and discussed to obtain consensus scores. Scores and their justification were then sent to project holders for feedback. Divergences (if any) were discussed by videoconference and the scores readjusted accordingly based on the data and justification provided. Disagreements only arose in cases where project holders and their partners had omitted to mention certain information during interviews. Final scores were generated at three levels: for each indicator, for each variable (mean), and for each project (synthetic performance index). “No data” indicators were removed from the calculation to avoid impacting the mean calculated for each variable and the synthetic index calculated for each project. A radar chart was drafted to synthesize the 3-level scoring completed for each project (Fig. 6B).

The results generated for each project therefore rely on both a semi-quantitative assessment based on scoring (database 2), and a qualitative analysis using abovementioned descriptors as well as the data on levers and barriers and solutions to barriers synthesized in database 3.

## Limitations of this study

### Limitations related to the use of a structured expert judgment method

Because it considers various dimensions (risk reduction, enabling conditions, and externalities) and aggregates different types of data, including qualitative data (e.g. related to the perception of funding conditions and NbCD co-benefits by project holders), this assessment uses a Structured Expert Judgment (SEJ) method based on a scoring system (see SM6). SEJ methods are increasingly used to assess adaptation, including in IPCC reports, as they have several advantages over conventional quantitative methods. They (1) allow to bring together multiple types of information, (2) describe climate change adaptation in a more comprehensive way, (3) reflect the diversity of context specificities, and (4) deliver results over short periods of time, which is crucial to support adaptation progress^87^.

However, SEJ also have limitations that must be thoroughly addressed to ensure the robustness of the results generated. These limitations are twofold. First, they relate to the design of the assessment method, that is, the choice of the variables and indicators. Two different teams involved in the design of a NbCD assessment would likely organize variables and indicators in a different way, making the general shaping of the results different. For example, our assessment method is different from the IUCN global standard for NbS (although some variables and indicators are common between the IUCN standard and our assessment protocol). Theoretically, such discrepancies can be reduced by building on previous methodological studies, which we did. This allows to generate results that can, to some extent, be compared with, or put into perspective, with the results of other studies. Second, the limitations of SEJ relate to the scoring exercise (i.e. attributing scores to indicators), which requires the implementation of strict rules. These rules are (1) to precisely define and describe each score level to support experts in both selecting the appropriate information to document indicators and attributing scores to indicators, and (2) to organize open expert exchanges around scoring to share difficulties (if any) and experience, and to clarify (especially in the scoring test phase) grey areas and areas of disagreement (if any). These two rules are key to lay robust methodological foundations and thereby ensure the consistency of the assessment across case studies. They were applied at two levels in this study, firstly between the two researchers involved in the initial scoring, and secondly between the researchers and the actors to whom the scores were submitted for feedback. The discrepancies occurring during phase 1 (scoring by researchers) were few and they were resolved by the refinement of the methodological notice when they were due to inaccuracies in the boundaries between two scores, and by discussions on the justification of scores (i.e. data considered) when they arose from a disagreement on score attribution. In phase 2 (submission of scores to actors), few disagreements arose, which were due to the omission of information sharing by actors during data collection. In this case, the missing information was included in the databases and used to revise the concerned score. The latter was discussed and validated by all experts (researchers and actors).

### Limitations related to the focus on nature-based coastal defense

Because this evaluation focuses on NbCD only, it does not allow to compare the effectiveness and levers and barriers to NbCD with the effectiveness and levers and barriers to other coastal adaptation options in studied territories. The way to overcome this limitation is to apply to same assessment protocol to other adaptation options, which is made possible by the fact that domains, variables and indicators are generic. This would make it possible to distinguish between generic levers and barriers applying to coastal adaptation measures in general and NbCD-specific levers and barriers.

## Electronic supplementary material

Below is the link to the electronic supplementary material.

Supplementary Material 1

Supplementary Material  2

Supplementary Material  3

## Data Availability

All data generated or analyzed during this study are included in this published article and the Supplementary Information files. Non-academic deliverables are available from the corresponding author on reasonable request.
