# Supplementary material for: Assessing nature-based coastal defense
Source: Sci Rep. 2025 May 14;15:16798. doi: 10.1038/s41598-025-96744-7 (PMC12078504; doi:10.1038/s41598-025-96744-7)
Supplement: Supplementary file 1 — Supplementary Material 1 [file 41598_2025_96744_MOESM1_ESM.docx]

**Supplementary Material**

**Assessing Nature-based Coastal Defense:**

**Application to French Overseas Tropical Island Territories**

**Supplementary Material 1.** Main physical and human features of French Overseas Tropical Island Territories

**Supplementary Material 2**. Nature-Based Coastal Defense projects implemented in French Overseas Tropical Island Territories

**Supplementary Material 3**. Synthesis of the results of Nature-Based Coastal Defense projects’ scoring

**Supplementary Material 4.** Detailed results of the assessment (see Excel file SM4)

**Supplementary Material 5.** Synthesis of the results per project

**Supplementary Material 6.** Assessment protocol (see Excel file SM6)

**Supplementary Material 7.** Main sections of the interview guide

**Supplementary Material 1.** Main physical and human features of French Overseas Tropical Island Territories.

This table present the general characteristics of these territories, which highlight their diversity.

| Territory | Total land area (km^2^) | Main physical and ecological features | | Main human features | | References |
| --- | --- | --- | --- | --- | --- | --- |
|  |  | Island type (prevailing type in bold) | Marine and coastal ecosystems | Main city (capital) and population (No./%) | Main economic activities |  |
| Pacific Ocean | | | | | | |
| New Caledonia | 18 575 km^2^ | High mountainous islands and reef islands | Coral reefs, mangroves, rocky coasts, beach-dune systems | Noumea  (Municipality: 94,235 / 34,7 %  Urban area: 182,341 / 67,1 %) | Nickel mining, agriculture, construction, services and industries | Garcin et al., 2016  INSEE, 2019  IEOM, 2018 |
| French Polynesia | 4 167 km^2^ | High mountainous islands and reef islands (both in the Tuamotu atolls and on the barrier reefs of mountainous islands) | Coral reefs, mangroves, rocky coasts, beach-dune systems | Papeete  (Municipality: 26,926 / 9 %  Urban area: 136,771 / 45,7 %) | Tourism, pearl farming, fishing, agriculture | Duvat et al., 2017  INSEE, 2017  IEOM, 2017  Madi Moussa et al., 2019 |
| Wallis and Futuna | 142, 4 km^2^ | High mountainous islands and reef islands | Coral reefs, mangroves, rocky coasts, beach systems | Mata Utu  (Municipality: 1,029 / 7,7 %  Urban area: 8,333 / 62 %) | Agriculture, fishing, handicrafts, trade, tourism and transportation | GREF, 2020  INSEE, 2018  INSEE, 2019  IEOM, 2019 |
| Indian Ocean | | | | | | |
| Reunion | 2 512 km^2^ | High mountainous island | Coral reefs, rocky coasts, beach-dune systems | Saint-Denis  (Municipality: 147,931 / 17,2 %  Urban area: 202,993 / 23,6 %) | Agriculture, agribusiness, farming, tourism, industry and handicraft | Duvat et al., 2016  INSEE, 2017  IEDOM, 2018 |
| Mayotte | 374 km^2^ | High mountainous island, reef islands | Coral reefs, rocky coasts, beach systems | Mamoudzou  (Municipality: 71,437 / 25,6 %  Urban area: 227,245 / 81,3 %) | Agriculture, fisheries, aquaculture, transportation, tourism | Jeanson, 2009  INSEE, 2017  IEDOM, 2018 |
| Caribbean region | | | | | | |
| Saint-Martin | 53 km^2^ (French side only; 93 25 km^2^ with Sint Maarten) | High mountainous island | Coral reefs (limited), mangroves, rocky coasts, barrier beaches, beach-dune systems | Marigot  (Municipality: 3,229 / 9 %) | Tourism, construction, agriculture and industry | Battistini and Hinschberger, 1994  INSEE, 2017  IEDOM, 2018 |
| Saint-Barthelemy | 25 km^2^ | High mountainous island | Coral reefs, mangroves, rocky coasts, barrier beaches, beach-dune systems | Gustavia  (Total pop.: 9,961) | Tourism and construction | Caron, 2011  INSEE, 2019  IEDOM, 2017 |
| Martinique | 1 128 km^2^ | High mountainous island | Coral reefs, mangroves, rocky coasts, beach-dune systems | Fort-de-France  (Municipality: 80,041 / 21,5 %  Urban area: 120,724 / 32,4 %) | Tourism, agriculture, agribusiness, construction and industry | INSEE, 2017  IEDOM, 2019 |
| Guadeloupe | 1 628 km^2^ | High mountainous islands | Coral reefs, mangroves, rocky coasts, beach-dune systems | Pointe-à-Pitre  (Municipality: 15,923 / 4,1 %  Urban area: 252,271 / 64,6 %) | Tourism, construction, industry, agriculture and agribusiness | INSEE, 2017  IEDOM, 2018 |

**References used to document FOTIT:**

Battistini, R., Hinschberger, F. Cordons et lagunes du littoral de Saint-Martin (Antilles Françaises): dynamique et problèmes d'aménagement, in: Maire, R., Pomel, S., Salomon, J.-N. (Eds.), *Enregistreurs et indicateurs de l'évolution de l'environnement en zone tropicale*. Presses Universitaires de Bordeaux, Bordeaux, pp. 331–344 (1994).

Caron, V. Contrasted textural and taphonomic properties of high-energy wave de- posits cemented in beachrocks (St. Bartholomew Island, French West Indies). *Sediment. Geol.,* 189–208. <https://doi.org/10.1016/j.sedgeo.2011.03.002> (2011)

Duvat, V. K. E., Magnan A., Etienne S., Salmon C., & Pignon-Mussaud C. Assessing the impacts of and resilience to Tropical Cyclone Bejisa, Reunion Island (Indian Ocean). *Nat. Hazards*, **83(1)**, 601–640. <https://doi.org/10.1007/s11069-016-2338-5> (2016).

Duvat, V.K.E., Salvat, B., Salmon, C. Drivers of shoreline change in atoll reef islands of the Tuamotu Archipelago, French Polynesia. *Global Planet. Change* **158**, 134–154. <https://doi.org/10.1016/j.gloplacha.2017.09.016> (2017).

Garcin, M., Vendé-Leclerc, M., Maurizot, P., Le Cozannet, G., Robineau, B., Nicolae-Lerma, A. Lagoon islets as indicators of rencent environmental changes in the South Pacific. The New Caledonian example. *Cont. Shelf Res.* **122**, 120-140. <https://doi.org/10.1016/j.csr.2016.03.025> (2016).

Jeanson, M. Morphodynamique du littoral de Mayotte : des processus au réseau de surveillance. Thesis, University of Dunkerque (2009).

INSEE (synthesis studies): <https://www.insee.fr/fr/statistiques>

IEDOM (Caribbean and Indian Ocean territories): <https://www.iedom.fr/iedom/statistiques/>

IEOM (Pacific territories): <https://www.ieom.fr/ieom/espace-statistiques/>

Madi Moussa, R., Fogg, L., Bertucci, F., Calandra, M., Collin, A., Aubanel, A., Polti, S., Benet, A., Salvat, B., Galzin, R., Planes, S., Lecchini, D. Long-term coastline monitoring on a coral reef island (Moorea, French Polynesia*), Ocean Coast. Manage*. **180**: 104928. <https://doi.org/10.1016/j.oceacoaman.2019.104928> (2019).

**Supplementary Material 2** – Nature-Based Coastal Defense projects implemented in French Overseas Tropical Island Territories

This table details the main characteristics of study projects which were part of the mapping of Nature-based Coastal Defense projects.

| Project No. (general and per region) | Location | Project name  Degree of technical mastering (experimental, mastered, transferred) | Date of implementation (and date of first action if any) | Main goal | Description of actions | Project holder | Funding source (and total cost if known) |
| --- | --- | --- | --- | --- | --- | --- | --- |
| PACIFIC OCEAN | | | | | | | |
| No.1  Pacific 1 | New Caledonia  Nouméa, Grande Terre | Mangrove and beach vegetation restoration at Tina Golf by SOS Mangroves  Experimental | 2017-2023 | -Reduce current to future coastal erosion  -Preserve mangroves  -Support social reintegration  -Raise awareness about mangrove related ecosystem services | -Planting of 8 hectares of mangroves and 150m of beach vegetation  -Reconnection of the mangrove to the sea  -Networking to promote capacity building  -Reintegration projects | SOS Mangroves association | Multiple funding sources, including mutual aid and volunteering (not estimated) |
| No.2  Pacific 2 | New Caledonia  Bourail, Grande Terre | Protection and restoration of the riparian vegetation of the Nera River  Experimental | 2023-2025 | -Support the resilience of coastal ecosystems and watersheds to climatic pressures by protecting and restoring riparian forests  -Reduce coastal climate and environmental risks | -Restoring riparian forests in partnership with farmers  -Training activities  -Awareness raising  -Promote knowledge development on forests | Wildlife World Fund (WWF) branch in New Caledonia | KIWA initiative and World Wildlife Foundation (WWF) (530,000€) |
| No.3  Pacific 3 | New Caledonia  Bourail, Grande Terre  Multi-site | Coastal vegetation restoration by the Bwärä Tortues Marines association  Transferred | 2006-today | -Reduce current to future coastal erosion  -Protect turtle nesting sites  -Reconnect marine and coastal ecosystems | -Coastal vegetation planting (13 hectares)  -Removal of car access to beaches  -Awareness raising  -Networking to promote capacity building | Bwärä Tortues Marines association (Bourail, Grande Terre) | Multiple funding sources, including mutual aid (not estimated) |
| No.4  Pacific 4 | New Caledonia  Touho, Grande Terre | Mangrove restoration by the Hô-üt association Transferred | 2022 (previous actions started in 2013) | -Reduce current to future coastal erosion and marine flooding  -Raise awareness about mangrove related ecosystem services | -Mangrove planting (150m and 2,500 m^2^)  -Awareness raising | Hô-üt association | International Union for Nature Conservation (IUNC), Pacific Regional Environment Programme (SPREP), volunteering (8,000€) |
| No.5  Pacific 5 | New Caledonia  Poindimié, Grande Terre | Mangrove and coastal vegetation restoration by the Popwadene association  Experimental + Transferred | 2020-2023 (first similar actions started in the 1980s) | -Reduce current to future coastal erosion  -Preserve a major cultural, feeder and recreational site | -Mangrove planting (9,000m^2^)  -Coastal forest restoration (300m)  - Awareness raising | Saint-Denis tribe  Popwadene association | EU BEST 2.0+ (51,391€) |
| No.6  Pacific 6 | French Polynesia  Moorea & Tahiti, Society Islands; Ahe & Tikehau, Tuamotu-Gambier Islands  Multi-site | Coral reef restoration by Coral Gardeners Transferred | 2017-20….. | -Preserve the reef ecosystem  -Preserve the ecosystem services provided by coral reefs, including the coastal protection service  -Improve people's standard of living by creating jobs and preserving marine resources (e.g. fish stocks) | -Large-scale coral transplantation (1 million coral cuttings by 2025)  -Awareness raising | Coral Gardeners Association | Donations, partnership, public funding, sponsoring (approx. 1.2M€/year) |
| No.7  Pacific 7 | French Polynesia  Hao Atoll, Tuamotu-Gambier Islands | Restoration of the ocean beach crest's native vegetation  Experimental | 2022-2023 | -Reduce coastal erosion and marine flooding  -Preserve biodiversity and remove invasive exotic species  -Promote local development through job creation and equipment purchases | -Coastal vegetation restoration through planting: 1650m, 3 spots  -Awareness raising  -Youth training | Municipality of Hao | National Office for Biodiversity + Municipality of Hao (179,527€) |
| No.8  Pacific 8 | French Polynesia  Anaa Atoll, Tuamotu-Gambier Islands | Restoration of the Tukuhora Priority Conservation Area Experimental | 2021-2024 | -Reduce cyclone-induced marine flooding  -Protect the primary forest from human pressures (airport, village extension).  -Strengthen the ecosystem services associated with this vegetation formation: medicinal, socio-economic and cultural | -Forest vegetation planting (400 m^2^)  -Sustainable forest management: fire prevention, removal of waste from the former landfill (3 m^3^), reduction of vegetation cuttings within the airport perimeter  -Awareness raising  -Reintroduction of the endemic pupu rega snail | Local association Pu tahi haga no Ganaa | National Office for Biodiversity + International Union for Nature Conservation (IUNC) (73,300€) |
| No.9  Pacific 9 | French Polynesia  Paea, Tahiti, Society Islands | Restoration of the native coastal vegetation, including sub-mangrove, coastal forest and salt meadow  Experimental | 2021-2023 | -Reduce current to future coastal erosion  -Preserve biodiversity  -Propose a standardized restoration protocol for degraded coastal areas | -Vegetation planting (5,000 m^2^)  -Awareness raising | Te Ora Naho (Fédération des Associations de Protection de l'Environnement), French Polynesian Government Research Delegation | EU BEST2.0+ (and volunteering and mutual aid) (60,000€) |
| INDIAN OCEAN | | | | | | | |
| Project No. (general and per region) | Location (Territory + site) | Project name and location  Degree of technical mastering (experimental, mastered, transferred) | Date of implementation (and date of first action if any) | Main goal | Description of actions | Project holder | Funding source (and total coast if known) |
| No.10  Indian Ocean 1 | Reunion Island  Saint-Paul | Rehabilitation of Cambaie Beach  Experimental | 2019-2023 | -Reduce current coastal erosion  -Restore turtle nesting sites | - Removal of exotic tree species  -Beach reprofiling  -Planting of forest species (300m)  -Awareness raising | Municipality of Saint-Paul and the National Forestry Office | State and municipality (125,000€) |
| No.11  Indian Ocean 2 | Reunion Island  Saint-Paul | Enhancement and restoration of the wooded coastal fringe of l'Hermitage-les-Bains  Experimental | 2017-2023 (first actions in 2003) | -Reduce current coastal erosion  -Strengthen biodiversity  -Raise public awareness on biodiversity preservation  - Improving the quality of life for residents and tourists  -Encourage soft mobility practices | -Removal of exotic tree species  -Beach nourishment and reprofiling  -Forest species planting (2,200m) on the upper beach and back beach  -Awareness raising  -Control of uses, including user path management | Saint-Paul municipality | EU (FEDER), municipality, community of municipalities (3,630,802€) |
| No.12  Indian Ocean 3 | Reunion Island  Étang-Salé | Rehabilitation of a turtle nesting site at Gouffre de l’Étang-Salé  Experimental | 2010-2013 (first actions in 2007) | -Reduce current to future coastal erosion  -Restore turtle nesting sites | -Removal of exotic invasive species  -Beach reprofiling  -Restoration of the coastal indigenous forest (9.25 hectares, 250m)  -Promotion of sustainable beach use | National Forestry Office | EU (FEDER), National Forestry Office (300,000€) |
| No.13  Indian Ocean 4 | Reunion Island  West coast  Multi-site | Rehabilitation of sea turtle nesting sites on the West Coast  Transferred | 2017-2022 (first actions in the 1990s) | -Reduce current to future coastal erosion  -Restore turtle nesting sites  -Raise public awareness about proper beach management  -Federate stakeholders concerned by beach preservation  -Improving the living environment | -Removal of exotic plant species  -Beach vegetation planting and restoration in several sediment cells  -Awareness raising  -Training and capacity building  -Knowledge strengthening | Center for the Study and Discovery of Marine Turtles (CEDTM) | EU (FEDER), Region, State (746,000€) |
| CARIBBEAN | | | | | | | |
| Project No. (general and per region) | Location (Territory + site) | Project name and location  Degree of technical mastering (experimental, mastered, transferred) | Date of implementation (and date of first action if any) | Main goal | Description of actions | Project holder | Funding source (and total cost if known) |
| No.14  Caribbean 1 | Martinique  Fort-de-France | Z’Ab marina Experimental | 2018-2026 | -Reduce wave height in the harbor basin by supporting the extension of the mangrove  -Demonstrating that Nature-based Solutions are compatible with economic activities | -Installation of a technical disposal encouraging vertical accretion in shallow waters through the increase of bottom roughness (bamboo fascines, wooden breakers, biodegradable nets), aimed at enabling mangrove extension over a 250m^2^ area  -Planting of mangrove if necessary | Community of municipalities (CACEM) | EU Life ARTISAN project (480,000€) |
| No.15  Caribbean 2 | Martinique  Le Lamentin | RÉCIPROCITÉ Experimental | 2016-2025 (first actions in 2013) | -Restoring back mangrove functionalities  -Promoting sustainable relationships between the environment, people and local economic activities | -Inventory of biodiversity  -Awareness raising among schoolchildren and educational initiatives  -Removal of exotic invasive species  -Reforestation of the back mangrove (10 hectares)  -Promoting eco-responsible practices among economic actors  -Enhancement and renaturation of an islet through cultural and educational actions  -Removal of end-of-life boats from the bay and promotion of sustainable mooring management  -Enhancement of the Longvilliers River (promoting sustainable management from the watershed to the sea) | Municipality of Le Lamentin | Life4BEST, OFB (French Office for Biodiversity), AFD (French Development Agency), municipality of Le Lamentin (1M€) |
| No.16  Caribbean 3 | Martinique  Sainte-Anne | Reducing coastal risks at Boucaniers Village  Experimental | 2012-20.. | -Reduce current to future coastal erosion  -Reduce future marine flooding | -Removal of groins exacerbating coastal erosion  -Experimentation with a technical solution using sandbags to reduce coastal erosion (failure)  -Restoration of the indigenous coastal vegetation  -Beach nourishment | Club Med (tourism company) | Club Med (not estimated) |
| No.17  Caribbean 4 | Guadeloupe  Clugny Beach, Anse Maurice, Anse à la Gourde Beach, Salines Beach  Multi-site | Carib Coast  Transferred | 2019-2023 (first action in 2007) | -Reduce current to future coastal erosion  -Generate and disseminate knowledge on sustainable beach management  -Create a network of experts on coastal risks and adaptation to climate change | -Production of a best practice guide on beach management  -Beach vegetation restoration (enclosures + planting)  -Awareness raising and knowledge sharing | National Forestry Office (ONF) | EU FEDER, National Forestry Office (ONF) (322,135€) |
| No.18  Caribbean 5 | Guadeloupe  Port-Louis | Renaturation of North Port-Louis coastal area  Transferred | 2020-2022 (first action in 2000) | -Reduce coastal erosion  -Reconnect the mangrove to the sea  - Support the emergence of a nature-centric vision of the coastal zone | -Hydrological and geomorphic studies  -Ecological inventories  -Coastal road closure and reorganization of vehicle parking and travel in favor of soft mobility  - Beach vegetation restoration (enclosures + planting) | National Forestry Office (ONF) | State, National Forestry Office (ONF) (264,400€) |
| No.19  Caribbean 6 | Guadeloupe  La Saline Beach,  Le Gosier | Experimental approach to soft coastline management Experimental | 2012-20.. (first action in 2007) | -Reduce barrier beach erosion to protect a biodiversity priority area (swamp)  -Demonstrate that Sargassum deposits are not necessarily and only detrimental  -Promote soft coastaline management | -Multidecadal shoreline change study and monitoring  -Coastal road closure and reorganization of vehicle parking and travel in favor of soft mobility  -Maintenance of Sargassum leaves on the beach and promotion of sustainable beach management practices and control of beach uses with the aim of supporting the re-establishment and growth of the indigenous vegetation | Le Gosier municipality, French Coastal Conservatory (CDL) | Le Gosier municipality, French Coastal Conservatory (CDL) (not estimated) |
| No.20  Caribbean 7 | Guadeloupe  Sainte-Anne | Reducing coastal risks at Caravelle Village  Experimental | 2014-20.. | -Reduce current to future coastal erosion  -Maintain beach attractivity to support beach tourism | -Shoreline and beach change study  -Removal of a pontoon obstructing alongshore sediment transfer | Club Med (tourism company) | Club Med (tourism company) (not estimated) |
| No.21  Caribbean 8 | Guadeloupe  Jarry  Multi-site | Ja-riv  Experimental | 2016-20.. | -Reduce current to future coastal flooding  -Preserve and restore biodiversity | -Diagnostic of illegal occupations of the Public Maritime Domain by private companies  -Initiation and follow-up of land release by private companies  -Restoration of released land plots (decontamination, decompaction, mangrove restoration)  -Raising awareness about the ecological functions of swamp areas (development of a coastal path and knowledge sharing) | French Coastal Conservatory (CDL) | BRGM (Bureau of Geological and Mining Research), DEAL of Guadeloupe (State environmental division), EU FEDER, Water Office (ODE), French Office for Biodiversity (OFB), TotalEnergies Foundation (9,250,000€) |
| No.22  Caribbean 9 | Guadeloupe  Pointe-à-Pitre (Petit Cul-de-Sac Marin) and Folle Anse harbor (Marie-Galante Island)  Multi-site | Adapt’Island Experimental | 2019-2024 (first action in 2013) | -Promote an innovative ecosystem-based climate adaptation strategy and disseminate innovative ecosystem restoration techniques  -Restore and protect marine and coastal ecosystems  -Restore ecological continuity between marine and coastal ecosystems  -Involve local stakeholders and the civil society and raise awareness about ecosystem functions | -Establishment of a technical and scientific strategy for the Grand Port of Guadeloupe  -Mangrove restoration (80,000m^2^) through the removal of exotic invasive species, soil decompaction and decontamination  -Establishment of eco-friendly anchoring in seagrass area  -Coral transplantation to support coral reef restoration in Petit Cul-de-Sac Marin | Grand Port of Guadeloupe (GPMG) | EU Life, Grand Port of Guadeloupe (4,726,970€) |
| No.23  Caribbean 10 | Guadeloupe  Saint-François | Plant’Aksyon  Mastered | 2021-2022 (first action in 2018) | -Reduce current to future coastal erosion  -Awareness raising  -Protect biodiversity (flora and turtles) | -Construction of plant nurseries and training of schoolchildren to take cuttings  -Restoration of the indigenous coastal vegetation (enclosures and planting) | Saint-François municipality, 3 schools involved in Educative Marine and Terrestrial Areas | EU Life4BEST (35,775€) |

**Supplementary Material 3** – Synthesis of the results of Nature-based Coastal Defense projects’ scoring

This table presents the results obtained for each project, using the assessment framework presented in SM4.

| Project  Variable/Indicator | | Pacific Ocean | | | | | | | | | Indian Ocean | | | | Caribbean region | | | | | | | | | | Mean |
| --- | --- | --- | --- | --- | --- | --- | --- | --- | --- | --- | --- | --- | --- | --- | --- | --- | --- | --- | --- | --- | --- | --- | --- | --- | --- |
|  |  | New Caledonia | | | | | French Polynesia | | | | Reunion | | | | Martinique | | | Guadeloupe | | | | | | |  |
|  |  | No.1 | No.2 | No.3 | No.4 | No.5 | No.6 | No.7 | No.8 | No.9 | No.10 | No.11 | No.12 | No.13 | No.14 | No.15 | No.16 | No.17 | No.18 | No.19 | No.20 | No.21 | No.22 | No.23 |  |
| 1. Context | 1.1 | 1 | 2 | 2 | 1 | 3 | 1 | 1 | 1 | 1 | 1 | 2 | 1 | 1 | 1 | 2 | 1 | 1 | 0 | 2 | 1 | 2 | 2 | 1 | **1.34** |
|  | 1.2 | 1 | 1 | 2 | 2 | 2 | 2 | 2 | 0 | 2 | 3 | 2 | 3 | 2 | 2 | 3 | 1 | 3 | 3 | 3 | 1 | 3 | 2 | 3 | **2.09** |
|  | 1.3 | 1 | 1 | 3 | 3 | 2 | 1 | 1 | 1 | 1 | 2 | 2 | 1 | 3 | 1 | 1 | 1 | 3 | 3 | 1 | 0 | 1 | 2 | 2 | **1.60** |
|  | 1.4 | 2 | 2 | 2 | 2 | 2 | 1 | 2 | 2 | 1 | 2 | 2 | 2 | 2 | 2 | 2 | 1 | 2 | 2 | 2 | 2 | 1 | 2 | 2 | **1.83** |
| 2. Governance | 2.1 | 2 | 3 | 3 | 3 | 3 | 2 | 1 | 1 | 3 | 3 | 1 | 3 | 3 | 3 | 3 | 1 | 2 | 3 | 2 | 1 | 2 | 1 | 3 | **2.26** |
|  | 2.2 | 2 | 3 | 3 | 3 | 3 | 2 | 3 | 2 | 3 | 2 | 2 | 2 | 3 | 2 | 3 | 1 | 2 | 3 | 1 | 1 | 2 | 2 | 2 | **2.26** |
|  | 2.3 | 2 | 2 | 2 | 2 | 2 | 2 | 1 | 1 | 2 | 2 | 2 | 1 | 3 | 1 | 3 | 1 | 2 | 1 | 1 | 1 | 2 | 2 | 2 | **1.73** |
|  | 2.4 | 2 | 3 | 3 | 3 | 3 | 3 | 3 | 1 | 3 | 3 | 2 | 2 | 2 | 3 | 3 | ND | 2 | 2 | 3 | 1 | 2 | 2 | 3 | **2.45** |
| 3. Funding | 3.1 | 1 | 1 | 1 | 1 | 1 | 1 | 1 | 1 | 0 | 1 | 1 | 1 | 1 | 1 | 1 | 1 | 1 | 1 | 1 | 1 | 1 | 1 | 1 | **0.96** |
|  | 3.2 | 1 | 3 | 2 | 2 | 3 | 3 | 2 | 1 | 1 | 3 | 3 | 3 | 3 | 2 | 2 | 2 | 2 | 2 | 3 | 2 | 2 | 3 | 2 | **2.26** |
|  | 3.3 | 1 | 2 | 2 | 2 | 3 | 3 | 1 | 2 | 2 | 2 | 2 | 1 | 2 | 1 | 2 | 2 | 2 | 2 | 3 | 2 | 3 | 3 | 3 | **2.09** |
| 4. Social acceptability | 4.1 | ND | ND | ND | 2 | 2 | 2 | 2 | 1 | 2 | 2 | 2 | 2 | 2 | 2 | 2 | 2 | 2 | 2 | 2 | 2 | 2 | 2 | 2 | **1.95** |
|  | 4.2 | ND | ND | ND | 3 | 3 | 3 | 3 | ND | 3 | 3 | 3 | 3 | 3 | 3 | 3 | 3 | 2 | 2 | 2 | 2 | 2 | 2 | 2 | **2.63** |
|  | 4.3 | 2 | 3 | 2 | 2 | 3 | 2 | 1 | 1 | 1 | 2 | 2 | 1 | 3 | 1 | 3 | 0 | 2 | 3 | 0 | 0 | 1 | 1 | 2 | **1.65** |
| 5. Technical effectiveness | 5.1 | 2 | 1 | 2 | 2 | 2 | 1 | 2 | 2 | 1 | 2 | 2 | 1 | 2 | 3 | 1 | 2 | 2 | 2 | 1 | 2 | 1 | 2 | 2 | **1.74** |
|  | 5.2 | 1 | 2 | 3 | 3 | 2 | 1 | 1 | 1 | 2 | 2 | 1 | 1 | 3 | 2 | 1 | 1 | 3 | 3 | 2 | 1 | 1 | 2 | 3 | **1.83** |
|  | 5.3 | 1 | 1 | 2 | 1 | 1 | 1 | 2 | 2 | 1 | 2 | 1 | 2 | 2 | 2 | 1 | 2 | 2 | 2 | 1 | 1 | 1 | 1 | 2 | **1.48** |
|  | 5.4 | 2 | 1 | 2 | 1 | 2 | 1 | 1 | 1 | 1 | 1 | 1 | 1 | 2 | 0 | 1 | 2 | 1 | 2 | 1 | 1 | 1 | 1 | 1 | **1.22** |
|  | 5.5 | 2 | 1 | 2 | 2 | 2 | 1 | 1 | 1 | 1 | 2 | 1 | 2 | 2 | 0 | 2 | 1 | 2 | 2 | 0 | 1 | 1 | 1 | 1 | **1.35** |
|  | 5.6 | 1 | 1 | 1 | 1 | 1 | 1 | 1 | 1 | 1 | 1 | 1 | 1 | 1 | 1 | 1 | 1 | 1 | 1 | 1 | 1 | 1 | 1 | 1 | **1.00** |
| 6. Studies, monitoring, evaluation | 6.1 | 1 | 1 | 1 | 0 | 0 | 1 | 0 | 1 | 1 | 0 | 1 | 1 | 2 | 3 | 2 | 1 | 1 | 2 | 0 | 1 | 2 | 2 | 0 | **1.04** |
|  | 6.2 | 1 | 2 | 2 | 2 | 2 | 2 | 2 | 2 | 2 | 2 | 2 | 2 | 2 | 2 | 2 | 1 | 1 | 2 | 2 | 1 | 2 | 2 | 2 | **1.83** |
|  | 6.3 | 0 | 2 | 1 | 0 | 1 | 2 | 1 | 1 | 0 | 1 | 1 | 0 | 2 | 2 | 2 | 0 | 2 | 1 | 0 | 0 | 1 | 2 | 2 | **1.04** |
| 7. Co-benefits & disbenefits | 7.1 | 1 | 1 | 1 | 1 | 1 | 1 | 1 | 2 | 1 | 2 | 1 | 2 | 3 | 1 | 1 | 1 | 1 | 1 | 1 | 0 | 1 | 1 | 1 | **1.17** |
|  | 7.2 | 3 | 3 | 3 | 2 | 3 | 2 | 2 | 2 | 2 | 2 | 2 | 2 | 3 | 1 | 3 | 1 | 2 | 2 | 2 | 1 | 3 | 2 | 2 | **2.17** |
|  | 7.3 | 1 | 1 | 1 | 1 | 1 | 1 | 1 | 0 | 1 | 2 | 1 | 2 | 1 | 1 | 1 | 0 | 1 | 1 | 1 | 0 | 1 | 1 | 1 | **0.96** |
|  | 7.4 | 3 | 2 | 2 | 3 | 3 | 2 | 3 | 2 | 3 | 3 | 1 | 3 | 3 | 3 | 3 | 2 | 2 | 2 | 1 | 2 | 2 | 2 | 2 | **2.35** |
| 8. Adaptive potential | 8.1 | 1 | 1 | 1 | 2 | 2 | 1 | 1 | 1 | 1 | 1 | 1 | 1 | 1 | 2 | 2 | 1 | 1 | 0 | 2 | 1 | 2 | 2 | 1 | **1.26** |
|  | 8.2 | 1 | 1 | 2 | 1 | 3 | 1 | 1 | 1 | 1 | 1 | 1 | 1 | 2 | 0 | 2 | 1 | 2 | 2 | 1 | 1 | 2 | 1 | 2 | **1.35** |
|  | 8.3 | 2 | 2 | 2 | 2 | 2 | 2 | 2 | 2 | 2 | 2 | 1 | 2 | 2 | 2 | 2 | 2 | 2 | 2 | 2 | 2 | 2 | 2 | 2 | **1.96** |
| Synthetic index (out of 100) | | 50.6 | 61.3 | 67.9 | 64.4 | 73.9 | 58.3 | 54.4 | 45.4 | 53.9 | 66.7 | 55.0 | 58.3 | 77.2 | 60.0 | 71.1 | 44.3 | 63.9 | 65.0 | 52.2 | 39.4 | 59.4 | 62.2 | 65.0 | **59.5** |

**Supplementary Material 4.** Detailed results of the assessment

See Excel file SM4

This table details scores obtained for each indicator, as well as their justification based upon the data collected for each project.

**Supplementary Material 5.** Synthesis of the results per project

The following radar charts were generated for each project and provide a sketch view of the results obtained, highlighting their strengths and weaknesses.

**Pacific Ocean (for project location and description, see SM2)**


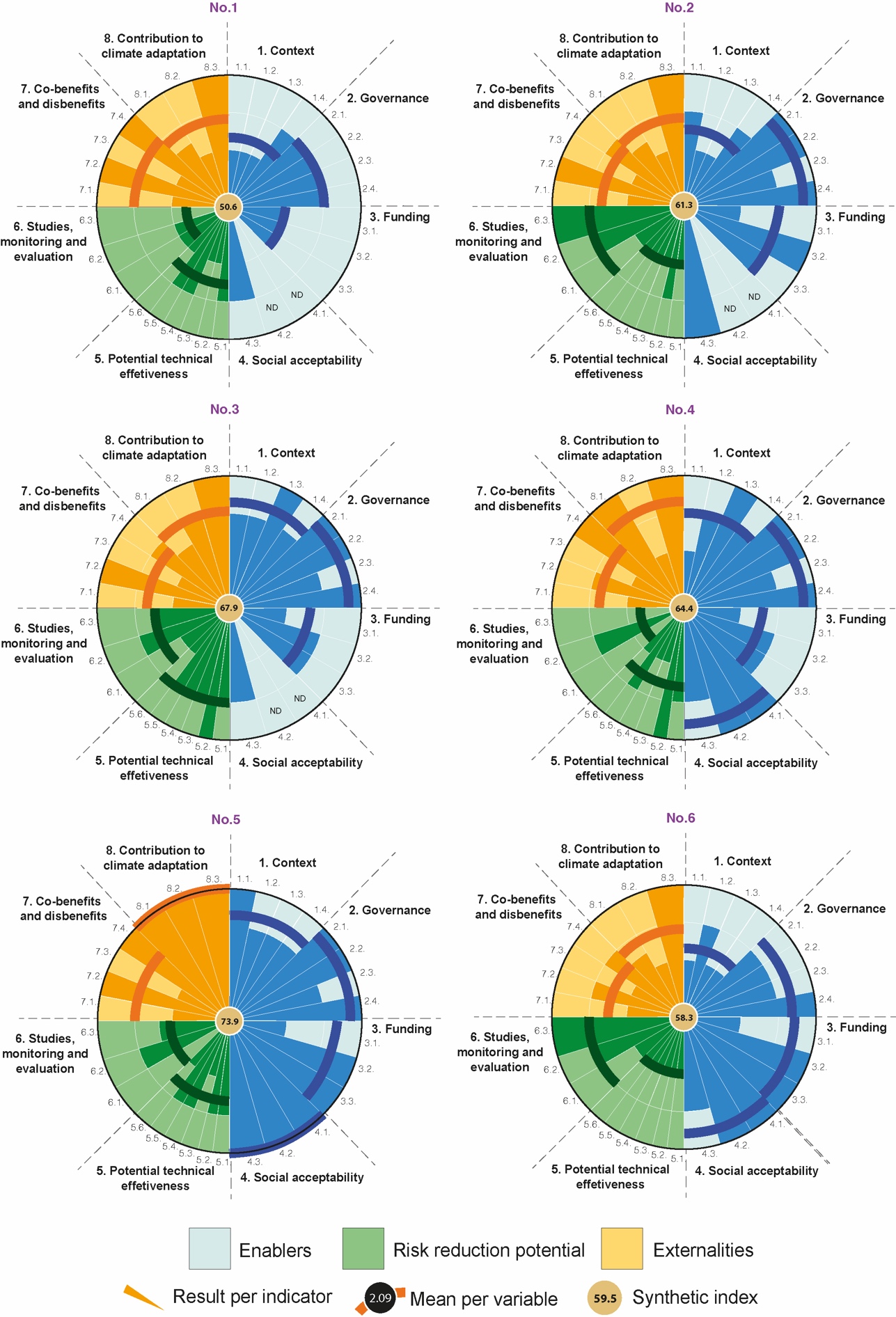


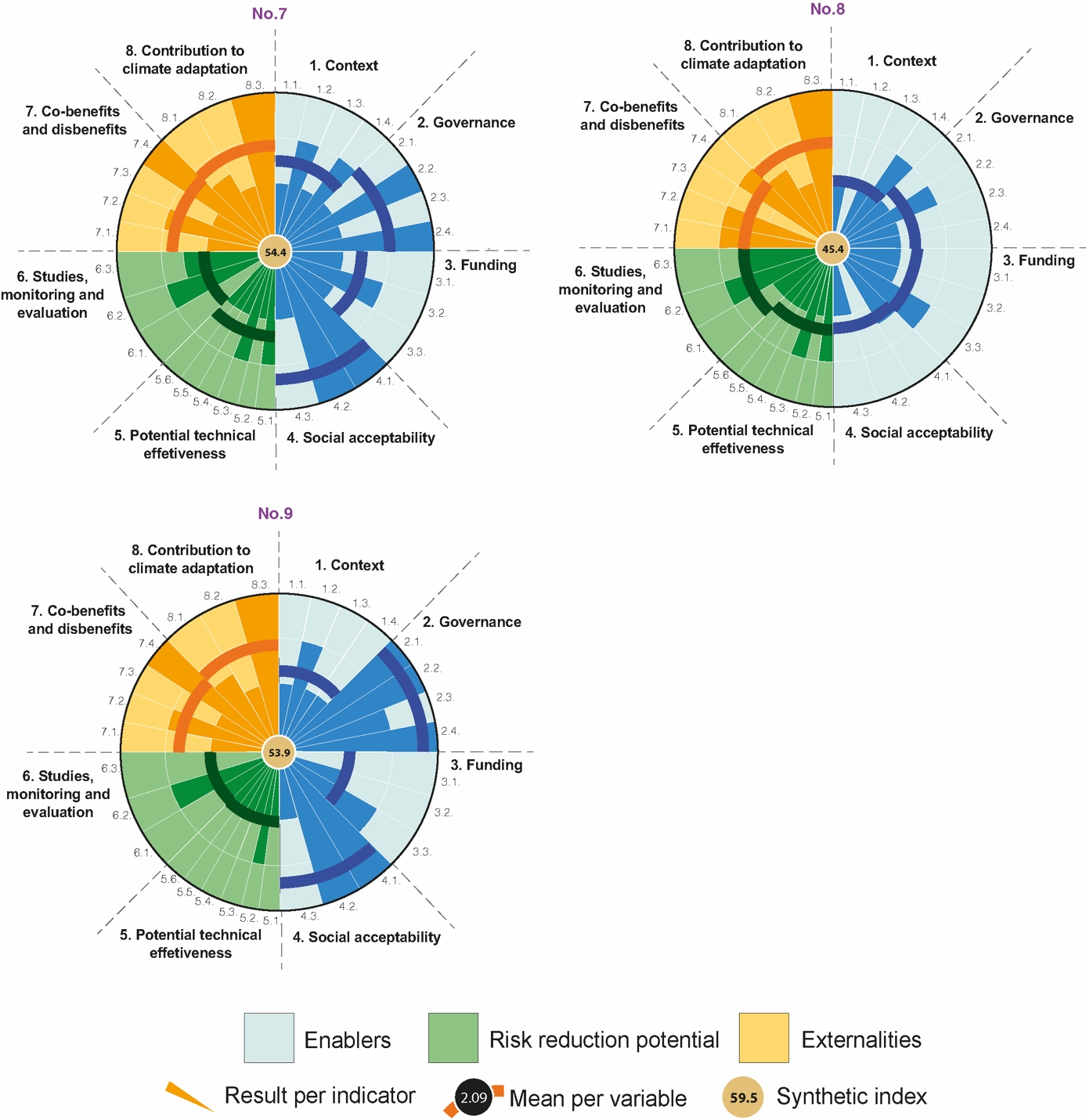


**Indian Ocean (for project location and description, see SM2)**


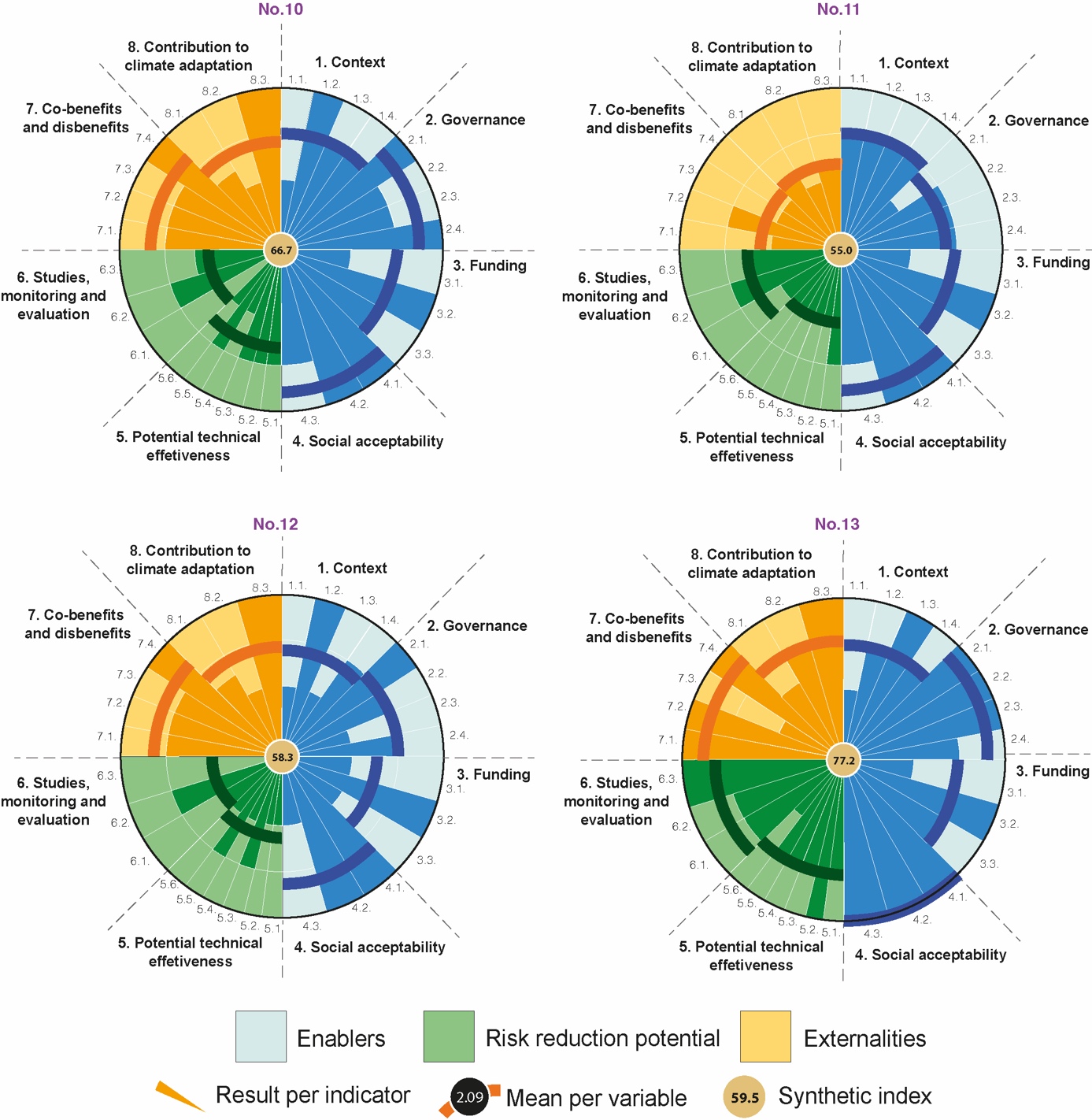


**Caribbean region (for project location and description, see SM2)**


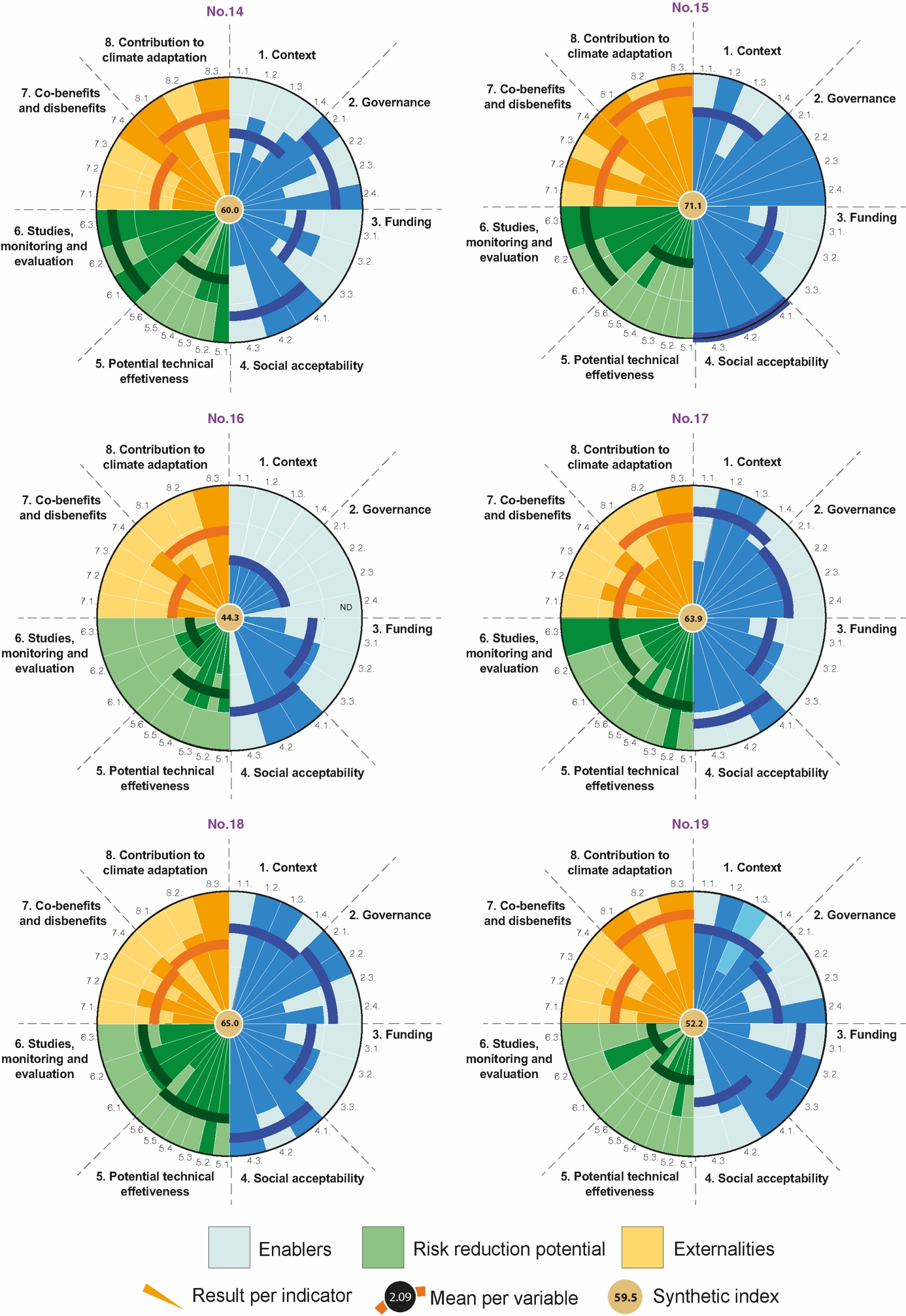


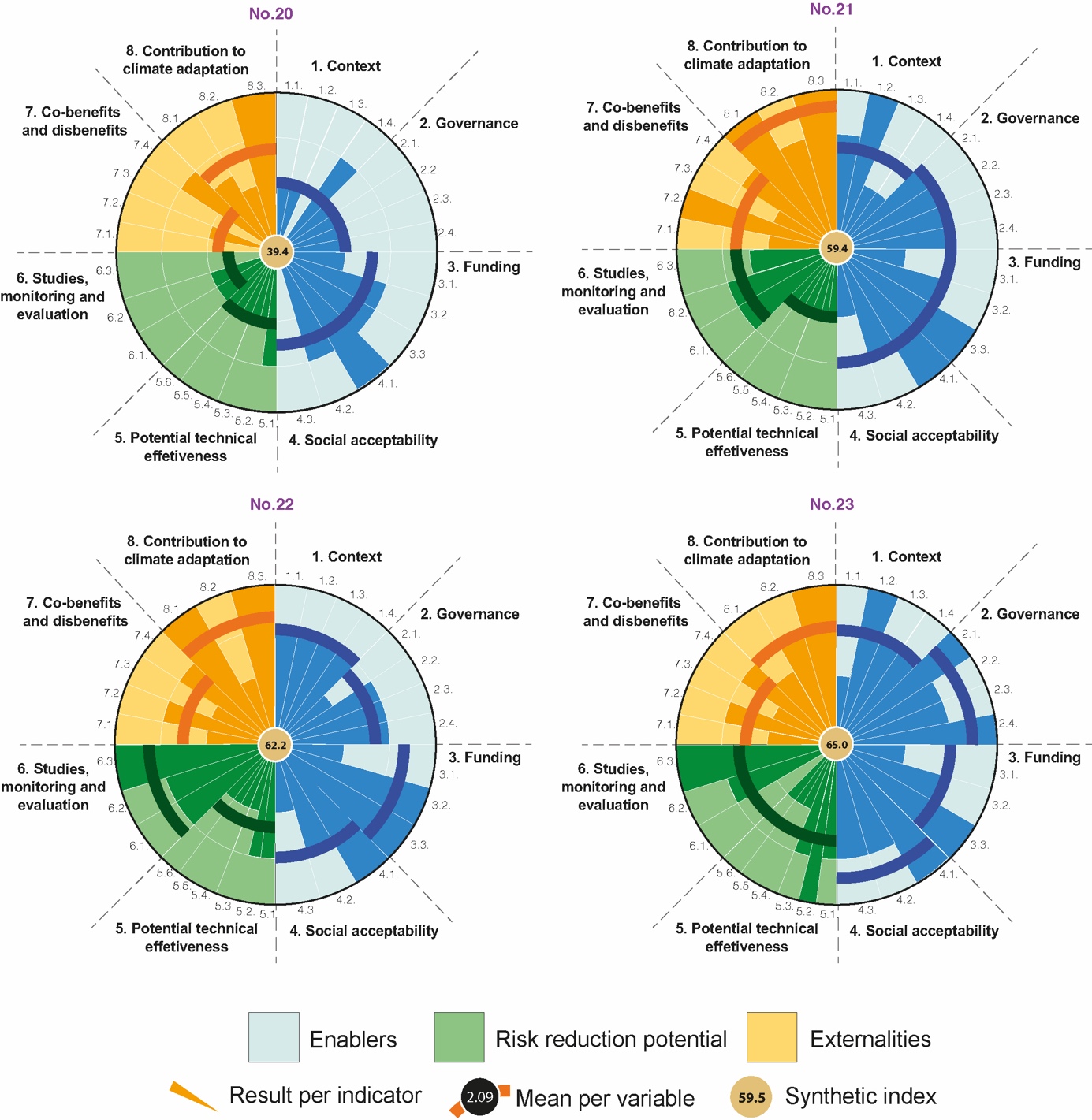


**Supplementary Material 6.** Assessment protocol

See Excel file SM6

This table details the method used to assess projects, including the domains (column A), variables (column B) and indicators (column D) considered. It presents the guiding questions associated with each variable (column C) and indicator (column E), as well as the scoring system (column F) and guidelines for scoring (column G).

**Supplementary Material 7.** Main sections of the interview guide.

This table summarizes the main sections (left column) and sub-sections (right column) of the interview guide used to document Nature-based Coastal Defense projects. The semi-structured interviews were conducted with project holders and partners.

| 1. Project’s location | Overseas territory |
| --- | --- |
|  | Island |
|  | Municipality/community |
|  | Site |
| 2. Origin of the project | Reasons for/context of design and implementation, initiator |
|  | Previous actions implemented at the same site to address coastal risks |
| 3. Context | Policies, regulations and tools applying in relation with the site and the project (local and national, if relevant) |
|  | Land tenure at project location and nearby |
|  | Technical readiness at the territory’s scale |
|  | Cognitive context at the territory’s scale |
| 4. Description of project | Project holder and partners (internal and external) and their respective roles in project implementation; collaborations and networking |
|  | Main and secondary goal(s) of the project, including the coastal risks targeted |
|  | Understanding of risk and how the project can reduce it by project holder and partners |
|  | Technical actions, and their spatial and temporal scales |
|  | Non-technical actions, and their spatial and temporal scales |
|  | Project’s governance and related modalities |
|  | Previous, emerging or expected conflicts/conflictual issues |
|  | Social acceptability, public society’s involvement in the project, and empowerment |
|  | Project’s cost, funding source(s), economic evaluation |
|  | Human and technical capacities of project holder and partners |
|  | Support provided by studies and monitoring, and project’s final evaluation |
|  | Strategic and adaptive dimension |
|  | Co-benefits and beneficiaries; disbenefits and losers |
|  | Lock-ins and maladaptive effects |
|  | Levers and barriers to implementation and success |
